# Use of Single Prolonged Stress to Model Post-traumatic Stress Disorder in Rodents: What We Found and Where to Next?

**DOI:** 10.2174/011570159X377560250629183749

**Published:** 2025-07-17

**Authors:** Keke Ding, Cunbao He, Shaojie Yang, Jingji Wang, Guoqi Zhu

**Affiliations:** 1 Center for Xin'an Medicine and Modernization of Traditional Chinese Medicine of IHM, and Key Laboratory of Molecular Biology (Brain diseases), Anhui University of Chinese Medicine, Hefei, 230012, China;; 2 Acupuncture and Moxibustion Clinical Medical Research Center of Anhui Province, The Second Affiliation Hospital of Anhui University of Chinese Medicine, Hefei, 230061, China

**Keywords:** PTSD, single prolonged stress, pathogenesis, drug development, rodents, signaling pathways, neural circuits

## Abstract

Post-traumatic stress disorder (PTSD) represents a grave and expansive mental illness, caused by experiencing or witnessing traumatic events that invoke profound feelings of helplessness, fear and anxiety. Reflecting the clinical features of PTSD, the single prolonged stress (SPS) model in rodents was developed to elucidate the pathogenesis and identify potential therapeutic interventions. This review aimed to deepen our understanding of the mechanisms and therapeutic methods for PTSD. We conducted a comprehensive literature search on PubMed and Web of Science using keywords such as “SPS”, “PTSD”, and “mechanisms”. Clinical and animal research, especially the exploration of the mechanisms and treatments, were included in this review. We identified a total of 327 articles. After removing duplicates and screening the full texts, we selected only 137 articles. Based on the literature, we examined the parallels and divergences between PTSD and the SPS model regarding symptomatic manifestations, affected brain regions, and molecular markers, demonstrating that the SPS model can effectively replicate PTSD-like behaviors in rodents. Guided by clinical research findings, we further synthesized the mechanisms by which SPS induces PTSD, focusing on the modulation of relevant signaling pathways and neural circuits. Additionally, we reviewed potential intervention strategies for PTSD using this model, encompassing both pharmacological and non-pharmacological therapies. This review offers significant implications for basic research rooted in the clinical characteristics of PTSD, suggesting that studies utilizing the SPS model could enhance our understanding of PTSD and aid in the identification of effective treatment strategies.

## 1 INTRODUCTION

The prevalence of post-traumatic stress disorder (PTSD) is estimated to be approximately 6-8% within the general population. However, this incidence demonstrates elevated rates reaching 35% among individuals exposed to severe traumatic stressors, including refugee populations, military veterans, and survivors of interpersonal violence [1, 2]. The American Psychiatric Association's Diagnostic and Statistical Manual of Mental Disorders, Fifth Edition (DSM-5; American Psychiatric Association, 2013), delineates four distinct clusters of PTSD symptoms: intrusive re-experiencing of traumatic events, avoidance of trauma-related stimuli, negative alterations in cognition and mood, and marked changes in arousal and reactivity [3]. Currently, the primary treatments for PTSD are centered on trauma-focused therapies, including trauma-focused cognitive behavioral therapy and prolonged exposure therapy [4]. Medications employed in the treatment of PTSD include selective serotonin reuptake inhibitors. However, it is imperative to exercise caution and avoid the use of benzodiazepines or other sedative-hypnotic medications, as they have been associated with an exacerbation of intrusive and dissociative symptoms over time [4].

Existing preclinical models of PTSD predominantly simulate the condition through the induction of physical and psychological trauma. These models encompass a variety of methodologies, including Pavlovian fear conditioning paradigm (using the combination of unconditioned and conditioned stimulations) [5, 6], stress-enhanced fear learning [7, 8], exposure to predators or predator odors [9], and physiological stressors such as underwater trauma [10]. Additionally, protocols incorporating restraints and multiple concurrent stressors are utilized to mirror the complex nature of PTSD. Single prolonged stress (SPS) constitutes a multi-modal traumatic stress paradigm that sequentially incorporates three distinct stressors: two hours of restraint, a 20-minute forced swim, and exposure to ether [11]. This comprehensive procedure elicits a profound stress response through the convergence of psychological, physiological, and pharmacological stimuli [11, 12]. Since its inception in 1997, the SPS model has been extensively employed by researchers to simulate characteristics of PTSD [13-17].

As a narrative review, this study aims to synthesize multidisciplinary evidence regarding the SPS model as a translational tool for PTSD research. We conducted systematic searches in PubMed and Web of Science using medical subject headings (MeSH) terms including “SPS”, “post-traumatic stress disorder”, and “mechanisms”. Our initial search yielded 327 records. Following duplicate removal and rigorous screening of titles, abstracts, and full texts based on our predefined criteria, 137 articles were ultimately included for comprehensive analysis. Inclusion criteria: (1) Studies utilizing SPS as a rodent PTSD model; (2) Investigations reporting mechanistic findings, behavioral outcomes, or therapeutic interventions; (3) Articles published in English. Exclusion criteria: (1) Review articles, commentaries, or editorials; (2) Studies employing non-SPS-based PTSD models; (3) Publications with insufficient experimental data or methodological details. Initially, we undertook a comparative analysis to delineate the analogous changes and discrepancies between PTSD patients and SPS animal models. Building upon this foundation, we synthesized and categorized the mechanisms and therapeutic strategies uncovered. Finally, we discuss the key issues that need to be addressed when using this model, aiming to provide translational insights for the treatment of PTSD.

## 2 COMPARISONS BETWEEN PTSD IN PATIENTS AND SPS-INDUCED CHANGES IN ANIMAL MODELS

### 2.1 Symptoms in Patients and Behaviors in Animals

Most symptoms associated with PTSD are extensively studied in animal models. These include an exaggerated startle response, disruptions in sleep and circadian rhythms, and the avoidance of trauma-related stimuli [18-22]. Although fear generalization (overgeneralization of fear) and impairment of fear memory extinction (a new learning process leads to the formation of extinction memories) are not explicitly included in the DSM-5 diagnostic criteria, they have been consistently observed in PTSD patients [23, 24]. These phenomena are also readily investigated in animal models, providing valuable insights into the underlying mechanisms of the disorder [15, 25, 26] (Table **1**). However, the presence or absence of nightmares, self-blame, or trauma-related thoughts can only be ascertained through patient self-reports, as these symptoms are inherently subjective and cannot be objectively measured in the same manner as other physiological responses [1].

One week post-modeling of SPS, behavioral phenotypes included heightened anxiety, increased arousal, and pronounced fear behaviors [27]. Concurrently, there was a notable decline in social interaction, recognition memory, and spatial memory. Importantly, following a re-stress episode, these animals exhibited exacerbated anxiety, disrupted arousal, and impaired fear extinction [28]. Specifically, animals subjected to the SPS protocol exhibit prolonged contextual freezing times, decreased duration and frequency of entries into the open arms in the elevated plus maze, diminished sucrose preference in the sugar-water preference test, and increased immobility during the forced swimming test. Especially, SPS-induced anhedonia and despair also indicate a certain degree of intersection between PTSD and depression [29]. Furthermore, SPS-exposed mice or rats are characterized by heightened defensive behaviors, exaggerated startle responses, and disrupted sleep patterns, indicative of significant stress-induced alterations in both behavior and physiology [15-17]. These behavioral and physiological alterations closely mirror the characteristic symptoms observed in PTSD patients, thereby validating the reliability of the SPS animal model as an effective analogue for studying PTSD [30, 31].

### 2.2 Brain Regions

Prior studies have demonstrated both functional and structural changes in the prefrontal cortex (PFC), amygdala, and hippocampus in individuals diagnosed with PTSD [32-36]. The hyperactivation of the amygdala has been found to inversely correlate with the activity of the medial prefrontal cortex (mPFC), a relationship that further aligns with the severity of PTSD symptoms. Notably, patients with PTSD often exhibit significantly elevated amygdala activation compared to control subjects, implying that PTSD is characterized by pronounced amygdala “hyperactivity” [37, 38]. Specifically, the “hyperactivity” observed in the amygdala may promote enhanced associative fear learning, leading to more robust and easily generalized fear associations that are notably resistant to extinction.

Patients with PTSD who present with symptoms of emotional detachment—such as depersonalization, derealization, and emotional numbness—demonstrate increased activation in the mPFC and rostral anterior cingulate regions [39, 40]. These findings suggest that various phenotypes of PTSD are associated with distinct alterations, including changes in amygdala activity and connectivity patterns. Consequently, it is crucial to consider the heterogeneity of the disorder when designing preclinical experiments related to PTSD [41].

Behavioral tests show that SPS can recapitulate most symptoms of PTSD. By analyzing the literature, SPS may mainly affect brain regions such as the amygdala, hippocampus, cortex (prefrontal cortex, ACC, insula), hypothalamus, *etc*. The brain regions involved in the SPS animal model are basically consistent with those of PTSD patients, further indicating that the SPS model is a good paradigm for PTSD research. For an example, animals subjected to SPS demonstrate a diminished excitatory tone and hypoactivity in the mPFC compared to control groups [42, 43]. This finding aligns with the observed reduction in mPFC activation in individuals suffering from PTSD (Table **1**). However, new technologies and methods such as brain functional imaging and single-cell sequencing are of great significance for exploring more involved brain regions.

### 2.3 Changes of Biochemical Indices

#### 2.3.1 BDNF

Veterans with PTSD exhibited lower plasma concentrations of brain-derived neurotrophic factor (BDNF) and impaired cognitive performance compared to healthy controls [44]. Similarly, decreased levels of BDNF in the hippocampus have been reported in animal models of SPS, consistent with findings in PTSD patients [15-17]. However, other animal models of PTSD have shown either increases or no changes in BDNF levels [45-47]. Notably, the direct administration of BDNF into the subgenual mPFC significantly reduced conditioned fear for up to 48 hours. These observations suggest that targeted supplementation of BDNF in specific brain regions may possess therapeutic potential for PTSD [45, 48].

#### 2.3.2 Inflammation

Blood levels of tumor necrosis factor-alpha (TNF-α), interleukin-1 beta (IL-1β), and interleukin-6 (IL-6) are frequently chronically elevated in patients with PTSD, indicative of heightened inflammation and a dysregulated immune response [49, 50]. Similarly, the SPS animal model also demonstrated increased expression levels of these pro-inflammatory cytokines in the hippocampus [16]. The elevated levels of pro-inflammatory factors observed following the SPS procedure may be attributed to the hyperactivity of the sympathetic nervous system rather than increased cortisol levels [51, 52].

#### 2.3.3 HPA Axis

The hypothalamic-pituitary-adrenal (HPA) axis and the sympathetic nervous system play critical roles in modulating the stress response. However, patients with PTSD exhibit an atypical pattern characterized by decreased basal cortisol levels and elevated catecholamine levels, which challenges the prevailing assumption of heightened stress hormone levels [53, 54]. The increased hypothalamic secretion of adrenocorticotropic hormone-releasing hormone, combined with reduced adrenal release of cortisol in PTSD patients, is accompanied by enhanced negative feedback inhibition of the HPA axis [35]. This dysregulation in cortisol levels and HPA axis function can significantly impact sleep quality, manifesting as fragmented rapid eye movement (REM) sleep and diminished slow-wave sleep in individuals with PTSD [55]. The SPS model induces mRNA expression of dorsal hippocampal glucocorticoid receptors and was originally developed to provoke a strong stress response. Initially termed the time-dependent stress sensitization model, SPS is characterized by a rapid enhancement of negative feedback within the HPA axis observed seven days post-induction [56] (Table **1**).

#### 2.3.4 Neurotransmitters

N-acetyl-aspartate (NAA) concentrations are notably reduced in the hippocampus of PTSD patients, accompanied by neuronal loss [57, 58]. Additionally, elevated levels of glutamate (Glu) in the hippocampus have been documented in these individuals. Specifically, the Glu/NAA ratio has been positively correlated with the severity of PTSD symptoms [59, 60]. Animal study has indicated that both acute and chronic stresses influence Glu neurotransmitter levels in the forebrain [61]. Research further demonstrates that NAA concentrations in the anterior cingulate cortex (ACC) of PTSD patients negatively correlate with the time elapsed since trauma. Elevated NAA concentrations in the ACC have been observed in PTSD patients with recent trauma compared to non-traumatized controls. Moreover, patients with recent-onset PTSD exhibit increased levels of creatine (Cr) in the left amygdala and heightened concentrations of myo-inositol (mI) in the right amygdala [62]. These findings suggest that traumatic stress may disrupt neuronal connectivity between the prefrontal cortex and the amygdala by altering the synthesis and degradation of compounds such as glutamate in the prefrontal cortex [61, 63]. One day following the SPS procedure, rats displayed anxiety-like behaviors and an extinction of fear memory, characterized by decreased activation of glutamatergic neurons and increased activation of GABAergic neurons in the basal amygdala (BLA). Ten days after the SPS procedure, the same rats exhibited heightened anxiety and impaired fear memory extinction, with both glutamatergic and GABAergic neuron activation increased in the BLA, and a trend toward elevated GABAergic neuron activation in the central amygdala (CeA). These findings suggest that the activation patterns of glutamatergic and GABAergic neurons differ between the early and late stages of traumatic stress [64].

## 3 ADVANCES ON THE MECHANISMS OF PTSD USING SPS RODENT MODELS

In recent years, significant advancements have been made in understanding the mechanism of PTSD by using SPS to simulate PTSD-like behavior in rodents. Current evidence suggests two primary pathogenic mechanisms underlying PTSD development. On the one hand, the changes of specific molecules may affect synaptic function and plasticity, cell death, and impairment of antioxidant defense systems, and BBB function. On the other hand, the endocannabinoid (eCB) system, corticotropin-releasing hormone (CRH) system, oxytocin system, locus coeruleus (LC) - norepinephrine (NE) system, and orexin system are also involved in the occurrence of PTSD.

### 3.1 Molecular Changes Affect Synaptic Function and Plasticity, Cell Death, and Antioxidant Defense System and BBB Function

#### 3.1.1 Impairment of Synaptic Function and Plasticity

**Fig. (1):**
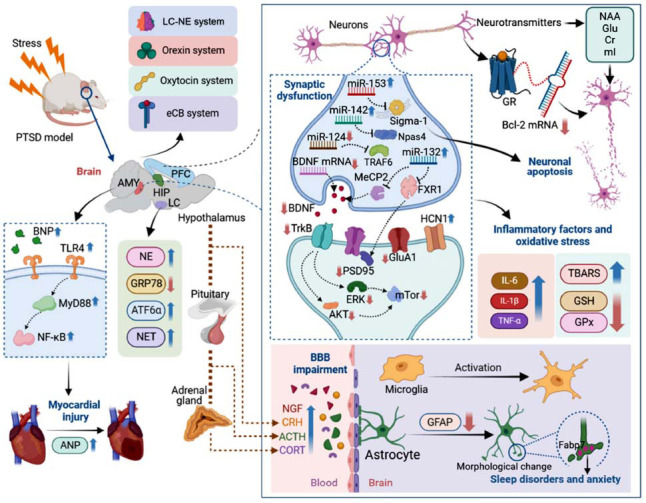
Mechanisms of PTSD revealed by single prolonged stress rodent model. Upon stress, the rats or mice displayed PTSD-like behaviors. Systemic reactions occur, including HPA axis, cardiac function, and significant abnormalities in molecular and neural circuit transmission in the brain, represented by oxyexin system, oxytocin system, and eCB system. Synaptic dysfunction is an important reason for fear and emotional dysfunction. In addition, inflammation and apoptosis are involved in the regulation process. Abnormalities in the blood-brain barrier are also common during the occurrence of PTSD. These collectively regulate PTSD symptoms. (**Abbreviations**: ACTH: adrenocorticotropic hormone, AKT: protein kinase B, AMY: amygdala, ANP: atrial natriuretic peptide, BBB: blood-brain barrier, BDNF: brain-derived neurotrophic factor, BNP: Brain natriuretic peptide, CORT: cortistatin, Cr: creatine, CRH: corticotropin relegating hormone, eCB: endocannabinoid, ERK: extracellular signal-regulated kinase, Glu: glutamate, GPx: glutathione peroxidase, GR: glucocorticoid receptor, GSH: glutathione, HCN1: hyperpolarization-activated cyclic nucleotide-gated 1, HIP: hippocampus, LC: locus coeruleus, mI: myo-inositol, mTOR: mechanistic target of rapamycin, NAA: N-acetyl-aspartate, NE: norepinephrine, NET: norepinephrine transporter protein, NF-kB: nuclear factor-kappa B, NGF: nerve growth factor, PFC: prefrontal cortex, TBARS: thiobarbituric acid reactive substances, TrkB: tyrosine kinase receptor B).

PTSD is conceptualized as a functional brain disorder, and synaptic dysfunction is the core mechanism of abnormal fear generalization or fear extinction. As reported, psychological stress elicited by SPS caused impairment of hippocampal synaptic transmission and synaptic plasticity, especially long-term potentiation [73, 74], further demonstrating that synaptic plasticity damage is an important mechanism in PTSD. In addition to electrophysiological results, SPS model rats or mice exhibited changes in synaptic structure and function, manifested as a decrease in the number of synapses and structural alterations [17]. At the molecular level, the hippocampus as well as the mPFC showed reduced expression of BDNF, p-TrkB and protein kinase B (Akt)- mechanistic target of rapamycin (mTOR) signal in SPS animal model [15, 16, 75-77] (Fig. **1**). Importantly, exogenous BDNF or activation of TrkB can improve SPS-induced synaptic and synaptic plasticity damage [48, 78]. Interestingly, the changes in those synaptic proteins may be regulated by epigenetic regulation of miRNAs [79-83], which implicates the specific miRNAs as potential therapeutic targets.

In addition to changes in synaptic structure and function, cellular ion channels can also affect the synaptic structure and function involved in PTSD-like behavior caused by SPS. Hyperpolarization-activated cyclic nucleotide-gated (HCN) ion channels are ubiquitously distributed throughout the brain and are abundantly expressed in various types of neurons. These channels play a pivotal role in modulating neuronal excitability, rhythmic neuronal activity, synaptic transmission, and synaptic plasticity, thereby mediating a multitude of physiological functions [84]. Experimental investigation has demonstrated that ZD7288, a selective blocker of HCN channels, significantly elevated BDNF protein in both the PFC and hippocampus, culminating in an anti-PTSD effect in the SPS-induced rat model [85]. Moreover, HCN channels are extensively distributed across functional regions of the forebrain, and the BDNF signaling pathway, which is associated with HCN1 and influences synaptic plasticity, may be implicated in the distinctive anti-PTSD-like effects observed with ketamine [86].

#### 3.1.2 Cell Death and Impairment of Antioxidant Defense Systems

In SPS animals, there is an upsurge in autophagy [69], oxidative stress, and inflammation within the hippocampus [70], all of which collectively promote apoptosis in this brain region [71]. Seven days post-SPS modeling, the amygdala exhibited increased apoptotic activity, along with alterations in intracellular calcium levels, evidenced by the upregulation of calmodulin and the downregulation of Ca^2+^/calmodulin-dependent protein kinase. These findings suggest that SPS disrupts fundamental cellular signaling mechanisms, potentially leading to hyperactivity and an elevated expression of fear [28]. Parallel pathophysiological alterations of neuronal apoptosis, autophagic activity, and calcium homeostasis were observed in the prefrontal cortex [87, 88]. Additionally, seven days following SPS modeling, a reduction in glutamate levels and an enhancement in glucocorticoid receptor (GR) expression were noted in the PFC, indicating a diminished excitatory tone in the mPFC [69, 89, 90].

In the SPS animal model, antioxidant defense systems, including reduced glutathione (GSH), glutathione peroxidase (GPx), and the reduced-to-oxidized glutathione ratio (GSH/GSSG), were observed to be diminished within the hippocampus [91]. Thiobarbituric acid reactive substances (TBARS), which are by-products of lipid peroxidation and serve as markers of oxidative stress, were found to be significantly elevated in the hippocampus of SPS animals [92, 93]. These findings indicate a reduction in antioxidant active components in the hippocampus of SPS models, underscoring the heightened oxidative stress in this region.

#### 3.1.3 BBB Impairment

Blood-brain barrier (BBB) disruption and microglial activation have been documented in the hippocampus of SPS rats. Notably, pretreatment with a microglial inhibitor, such as minocycline, not only attenuated microglial activation but also mitigated BBB degradation [94]. Astrocyte dysfunction has been implicated in the etiology of stress-related neuropsychiatric disorders [94]. It has been demonstrated that SPS reduces the expression of glial fibrillary acidic protein (GFAP), a key astrocytic marker, and induces morphological alterations in astrocytes within the hippocampus [15]. Furthermore, mutations in Fabp7, a lipid signaling molecule expressed by astrocytes, have been shown to influence stress-dependent sleep disorders and concomitant alterations in anxiety behaviors [27].

### 3.2 Changes of Specific Neural Systems

While PTSD affects a wide array of neurobiological systems, this review focuses on five well-characterized circuits: the eCB, CRH, oxytocin, LC–NE, and orexin systems. These systems were selected based on their consistent involvement in both SPS-induced behavioral phenotypes and clinical PTSD; central roles in fear conditioning, stress hormone regulation, memory extinction, arousal, and social cognition; and amenability to pharmacological modulation in rodent models. Other systems, such as dopamine and serotonin, were excluded due to limited SPS-specific evidence or overlapping with generalized stress responses (Fig. **1**).

#### 3.2.1 eCB System

According to animal models, the eCB system is involved in the occurrence of PTSD, particularly mediating stress responses, emotional changes, and traumatic memory [95]. The projection of mPFC to basolateral amygdala neurons can regulate the impact of eCBs on the extinction of fear memory, emphasizing the important role of eCBs in fear-related diseases, especially PTSD [96]. Using the SPS model, Xie *et al*. found that cannabidiol (CBD) can promote the expression of anandamide and hippocampal cannabinoid type 2 receptors (CB2Rs), inhibit central inflammation, and improve symptoms of PTSD [97]. Administration of endocannabinoid (eCB)1/2 receptor agonists has been shown to prevent alterations in BDNF expression and ameliorate depression-like symptoms in a rat model of PTSD, in addition to enhancing endogenous cannabinoid neurotransmission [98]. These findings collectively suggest that the eCB system represents a promising therapeutic target for addressing the mental and cognitive dysfunctions associated with PTSD.

#### 3.2.2 CRH System

Corticotropin-releasing hormone (CRH) - screening neurons are preferentially located in the paraventricular nucleus of the hypothalamus, primarily responsible for promoting the synthesis and release of adrenocorticotropic hormones from the pituitary gland. However, CRH is also present in other tissues, such as the amygdala and hippocampus. CRH receptors are classified into two types: corticotropin-releasing hormone type I receptor (CRHR1) and corticotropin-releasing hormone type II receptor (CRHR2), both of which play crucial roles in regulating the stress response. Research has shown that after SPS modeling, the expression of CRH and CRHR1 in the amygdala significantly increases, and the use of CRHR1 inhibitors can promote the extinction of fear memories [99, 100]. Tillinger *et al*. found that SPS promotes CRH expression in PVN, while CRH expression in the amygdala is not affected [101]. Wang *et al*. showed that SPS can cause a decrease in CRH expression in the hippocampus [102]. Wang *et al*. found that SPS can promote CRHR1 in the amygdala and mPFC, but not affect CRHR2 [103]. Therefore, further research is needed to investigate the changes and roles of CRH in various brain regions during the occurrence of PTSD.

#### 3.2.3 OXT System

SPS can promote the binding of hippocampal oxytocin to its receptors, suggesting that this process may be involved in stress-related memory changes [12]. However, subsequent research suggests that the repeated nasal administration of OXT in the early post-traumatic period not only prevents the onset of PTSD symptoms, but also enhances pro-social behavior [104]. It was observed that the mRNA and protein levels of OXTR were diminished in the mPFC and amygdala of SPS animals. Intranasal administration of OXT was found to restore the levels of OXTR in the mPFC and amygdala, an effect that was blocked by the OXT antagonist atosiban [105]. This administration of OXT also reinstated SPS-impaired pro-social behavior and OXTR expression in these brain regions [103]. It is worth noting that 83% of susceptible rats showed a resilient phenotype after undergoing oxytocin-based emotional remodeling, and SPS-induced morphological changes in the forelimb cortex and basolateral amygdala were eliminated by oxytocin [106]. Contrary to the above researches, intraperitoneal injection of oxytocin can inhibit fear extinction in normal animals, but does not affect fear extinction in SPS [107]. This study suggests that caution should be exercised when using oxytocin to treat PTSD. Therefore, a comprehensive investigation into the roles of the OXT system in mediating trauma-induced alterations in social behaviors is warranted.

#### 3.2.4 LC-NE System

Employing the SPS model, Li *et al*. conducted investigations into locus coeruleus mineralocorticoid receptor (MR) and glucocorticoid receptor (GR) expression in PTSD rats [108]. This study is based on the possibility that the LC may be involved in the release of corticosterone and the occurrence of PTSD. The results showed that MR and GR exhibited dynamic changes at different time points after SPS modeling [108]. SPS rats demonstrated marked anxiety-like behavior, with their ER stress response leading to neuronal apoptosis in the nucleus ambiguus, elevated levels of NE in the brain, and increased expression of tyrosine hydroxylase in the LC [109, 110]. These experimental findings suggest that abnormal activity of the LC-NE system may be implicated in the development of PTSD-like symptoms [109]. Noradrenergic neuronal signaling is also predominantly regulated by the norepinephrine transporter protein (NET), which belongs to the family of sodium chloride neurotransmitter transporter proteins. The highest concentrations of NET in the brain are found in the LC, and it is a primary target of many antidepressant drugs. However, the pattern of NET mRNA expression changes in the LC of SPS animals was not entirely consistent. Approximately half of the subjects exhibited an increased trend, whereas the other half showed levels similar to or lower than those of non-stressed controls. It has been posited that this variability may stem from differences in the degree of hyperanxiety triggered by SPS [111].

#### 3.2.5 Orexin System

Orexin neurons are mainly located in the lateral hypothalamic (LH), and by secreting orexin, they affect brain areas such as the hippocampus and amygdala to regulate cognitive function. Salehabadi *et al*. found that amygdala orexin receptor 1 (OX1R) can regulate memory acquisition and extinction, while inhibiting OX1R in the amygdala can improve PTSD [112]. Han *et al*. found that SPS down-regulated hypothalamic orexin-A, while increasing hippocampal OX1R and OX2R. Injection of orexin-A into the lateral ventricle improved spatial memory and appetite in SPS rats, and partially reversed the increase in OX1R and OX2R levels in the hippocampus and hypothalamus [113].

## 4 PRECLINICAL DRUG RESEARCHES USING THE SPS MODEL

### 4.1 Chemical Drugs

#### 4.1.1 N-methyl-D-aspartate Receptor (NMDAR) Modulators

**Fig. (2):**
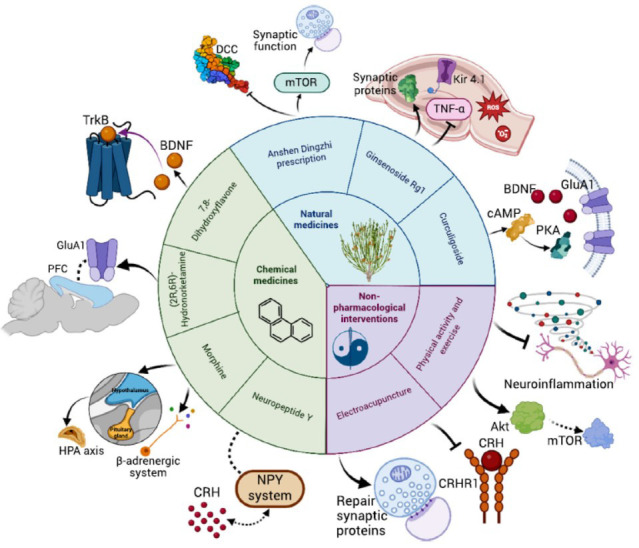
Available treatments for PTSD and underlying mechanisms. The treatments include three aspects, *i.e.* chemical medicines, natural medicines and non-pharmacological interventions. These treatments might involve the disclosed mechanisms to exert the effects. (**Abbreviations**: Akt: protein kinase B, BDNF: brain-derived neurotrophic factor, cAMP: cyclic adenosine monophosphate, CRH: corticotropin relegating hormone, DCC: deleted in colorectal cancer, HPA: hypothalamic-pituitary-adrenal, mTOR: mechanistic target of rapamycin, PFC: prefrontal cortex, PKA: protein kinase A, TNF-α: tumor necrosis factor-alpha, TrkB: tyrosine kinase receptor B).

NMDAR modulators have garnered significant attention in the search for effective treatments for PTSD. Systemic administration of a GluN2B-specific NMDAR antagonist, such as ifenprodil, has been shown to influence the retention of fear extinction learning [114]. A recent phase II clinical trial has highlighted the therapeutic potential of the NMDAR modulator NYX-783, demonstrating its efficacy in alleviating symptoms in patients with PTSD. Notably, NYX-783 exhibited no psychotropic side effects, a significant advantage over other rapid-acting antidepressants like ketamine [42]. Ketamine itself is known for its rapid and sustained antidepressant effects and has shown promising therapeutic outcomes in the treatment of PTSD [115]. However, its clinical application is hindered by side effects such as dissociative hallucinations and the potential for addiction [116]. Ketamine undergoes rapid metabolism *in vivo*, resulting in the formation of (2R,6R)-hydronorketamine (HNK), an active metabolite with similar receptor binding properties. Interestingly, the concentration of HNK in the brains of female mice is three times higher than that in male mice, which may account for observed sex differences in the antidepressant efficacy of ketamine. HNK mitigates chronic stress-induced negative mood states by restoring impaired AMPAR-dependent glutamatergic transmission [117] (Fig. **2**, Table **2**).

#### 4.1.2 Opioid Drugs

Morphine has been demonstrated to ameliorate deficits in anxiety-like behavior and dendritic morphology in the mPFC induced by SPS [126]. Intriguingly, the therapeutic efficacy of morphine administered immediately after stress exposure was less pronounced compared to administration 24 hours later. This finding suggests a temporal component to the drug's effectiveness. Researchers also noted that alterations in the HPA axis and central β-adrenergic activity could potentially influence the protective effects of morphine on PTSD-like symptoms. The regulatory processes governed by endogenous opioids under stress conditions appear to interact with the HPA axis in a time-dependent manner, the specifics of which remain to be fully elucidated [126]. Although opioids such as morphine have shown preclinical efficacy in SPS models, their clinical use in PTSD remains controversial due to two key issues. First, the high degree of comorbidity between PTSD and opioid use disorder (OUD) [133, 134], impedes effective diagnosis, assessment, and early intervention. A study suggested that approximately 1/3 of patients with OUD meet diagnostic criteria for PTSD [135]. Second, the paradoxical effect that short-term opioid use reduces acute distress, but long-term use exacerbates PTSD symptoms through dysregulation of mu-opioid receptors. A study reported that veterans with OUD and PTSD may be more effectively treated with trauma-focused, evidence-based psychotherapies, such as cognitive processing therapy (CPT), than with medication [136]. Also, the study has found the presence and potential increase in PTSD symptoms, depression, and anxiety in COVID-19 patients on long-term opioid substitution therapy (OST) [137].

#### 4.1.3 Others

Aripiprazole has been shown to ameliorate SPS-induced deficits in fear memory and to reduce dopamine efflux from the amygdala [127]. Notably, anxiety and depressive behaviors observed in the animals one week post-SPS exposure were reversed by the intranasal delivery of neuropeptide Y (NPY) [124]. Furthermore, researchers discovered that 21-day intraperitoneal injections of edaravone improved memory impairment and mitigated stress-induced disruptions in hippocampal antioxidant mechanisms, specifically normalizing the ratio of reduced GSH to oxidized GSH [125]. Utilizing the SPS model, differences were identified in the fecal microbial composition and cecal metabolites between animals susceptible to stress and those more resistant. Susceptible rats exhibited approximately 25% lower levels of cecal acetate, with a strong inverse relationship to anxiety indices [138]. Oral administration of acetate demonstrated a protective effect against SPS-induced functional changes by modulating neuronal and metabolic pathways [139]. Additionally, G1, an agonist of G protein-coupled estrogen receptor 1, exhibited protective effects on SPS-induced mitochondrial and synaptic dysfunctions in mice [140]. 7,8-Dihydroxyflavone (7,8-DHF) has been found to effectively mitigate PTSD-like symptoms, potentially through the targeting of TrkB receptors, thereby preventing astrocytic and synaptic deficits in the hippocampus [15].

### 4.2 Natural Medicines

Ginsenoside Rg1, known for its neuroprotective effects against various neurological disorders, has been demonstrated to reverse SPS-induced changes and alleviate anxiety-like behavior [16]. Systemic administration of curculigoside (CUR) improved PTSD-related behaviors and hippocampal synaptic deficits induced by SPS, by reducing the expression of proteins such as BDNF, TrkB, GluA1, and Arc, which are implicated in synaptic damage [17]. Importantly, cAMP-PKA signaling also takes part in the fear extinction [141, 142], and this mechanism might explain the effect of CUR. Puerarin was observed to attenuate anxiety-like behavior without affecting motor activity in SPS-exposed mice. The mechanism underlying this effect involves an increase in SPS-induced progesterone and isoprogesterone levels in the brain or serum, and a reduction in CRH levels [143]. Puerarin was also noted to alleviate PTSD by regulating the expression of key genes (CD36, CD59, HBS1L, and DYNC1H1) and metabolites (elaidic acid, daidzein, 3-succinoylpyridine, and 5-(2,5-dihydroxyhexyl) oxolan-2-one), as well as modulating critical pathways such as antigen processing and presentation, synthesis and degradation of ketone bodies, and the one-carbon pool by folate [120]. Tanshinone IIA administration resulted in reduced anxiety-like behavior and fear memory abnormalities in SPS animals. This effect was mediated through the activation of the cAMP response element-binding protein (CREB)/BDNF/TrkB signaling pathway in the hippocampus, which led to the upregulation of synapse-associated proteins [121]. Recent study has also highlighted the therapeutic potential of diosgenin in stress-induced depression [144]. Diosgenin modulated adenosine and its metabolites in various brain regions and normalized elevated serum corticosterone levels in SPS mice [145]. Furthermore, intraperitoneal injection of salidroside for 14 consecutive days in SPS rats exhibited neuroprotective effects by inhibiting hippocampal neuronal apoptosis and the activation of the NF-κB/iNOS/COX-2 signaling pathway. This, in turn, reduced the release of inflammatory factors (TNF-α and IL-1β) and the activation of microglial cells, thereby alleviating anxiety-like behaviors and memory impairment in SPS rats [146].

Withania somnifera (WS) Dunal has been shown to prevent memory impairment induced by PTSD and to reverse oxidative stress system alterations caused by SPS. Mechanistically, WS appears to confer memory protection by safeguarding the antioxidant mechanisms within the hippocampus [122]. Similarly, Korean Red Ginseng (KRG), an herbal supplement, was administered *via* intraperitoneal injection at doses of 20, 50, or 100 mg/kg/day for 14 consecutive days to rats modeled with SPS. The administration of KRG significantly attenuated cognitive and spatial memory deficits and enhanced the expression of BDNF mRNA and PSD-95 in the hippocampus [76, 118].

The Anshen Dingzhi prescription (ADP) stands as a pivotal formula in the treatment of panic and sleep disorders [23]. Previous studies have illustrated that oral administration of 36.8 mg/kg ADP for 14 consecutive days effectively prevents PTSD-like behaviors. The underlying mechanisms likely involve the modulation of synaptic and mitochondrial function, as well as the regulation of the deleted in colorectal cancer (DCC) in the hippocampus [23, 147, 148]. Polysaccharides from *Polygonatum sibiricum* (PSP) exhibit significant antidepressant efficacy [149, 150]. Experimental evidence from our group demonstrates that PSP can prevent SPS-induced fear memory generalization in mice, and the mechanism may be related to its effects on antioxidant, anti-inflammation, and synaptic repair [24].

### 4.3 Non-pharmacological Interventions

#### 4.3.1 Acupuncture (EA)

Electroacupuncture (EA) has been shown to reverse elevated levels of corticotropin-releasing hormone (CRH) and its receptor CRHR1 expression in the amygdala, thereby ameliorating fearful and anxious behaviors in SPS-exposed mice [100]. Additionally, EA enhances long-term potentiation in the hippocampus, repairs synaptic morphology, increases BDNF levels in both the amygdala and hippocampus, and upregulates the expression of SYN, GAP43, and PSD-95 in these regions [151-153]. Early intervention with EA has been found to attenuate SPS-induced anxiety and depression-like behaviors and partially normalize lipid alterations in the hippocampal region [129]. SPS disrupts the activation of the ventromedial prefrontal cortex (vmPFC) and its inputs to the ventral tegmental area (VTA), which may contribute to anxiety-like behaviors. EA has been shown to repair pathological changes in the vmPFC and its projections to the VTA, suggesting a potential mechanism underlying its therapeutic effects in PTSD [128, 130].

#### 4.3.2 Transcranial Photobiomodulation (PBM)

PBM is an emerging non-invasive neurological function modulation technology that regulates mitochondrial respiration within the cerebral cortex in a non-destructive and non-thermal manner by means of low-power, high-throughput near-infrared light, which in turn enhances the brain's oxygen supply and metabolic efficiency and achieves the effect of improving brain function [154, 155]. Studies have reported that early CW 808 nm laser irradiation through the skull could treat PTSD [156, 157]. By comparing the effectiveness of pulsed 810 nm laser transcranial irradiation (10 Hz) with CW 810 nm laser irradiation for the treatment of PTSD, the researchers have found that pulsed 810 nm laser phototherapy (P-PT) reversed the SPS program-induced increase in c-fos expression in rat mPFC. In contrast, CW-PT had no effect on c-fos expression and did not improve anxiety-like behavior in stressed rats. Thus, P-PT has the potential to be a new non-invasive treatment for PTSD [158].

#### 4.3.3 Hyperbaric Oxygen Therapy (HBOT)

HBOT is considered a safe and commonly used medical treatment. In recent years, there has also been much confirmation of the role of this non-pharmacological therapy in activating brain regions and protecting neurons. Findings suggest that HBOT restored SPS-impaired fear extinction retrieval, attenuated anxiety-like behaviors, and reversed IL monoamine efflux levels and corticosterone profiles [131].

#### 4.3.4 Physical Activity and Exercise

Physical activity and exercise have attracted widespread attention in improving anxiety, especially in the prevention and treatment of PTSD. In terms of treatment, the resistance exercise through climbing a 1-m-high ladder 15 times can improve SPS induced PTSD like behavior, which may be related to regulating the Akt/mTOR pathway and neuroinflammation [159].

## 5 FUTURE PROSPECTIVE

There are still three important aspects that warrant further investigation regarding the application of the SPS model. Firstly, there are still certain symptom clusters of PTSD that cannot be replicated by SPS models, and the modeling process or experimental methods require refinement. Secondly, the susceptibility and resistance of SPS models are key research methods in the future and require further investigation. Finally, based on the revelation of susceptibility and resistance mechanisms, developing intervention strategies from a prevention perspective is of great significance for stress-exposed populations.

In this review, we systematically analyzed the similarities and differences in the pathological changes between the SPS animal model and PTSD patients. The SPS animal model can replicate key behavioral changes and physiological features of PTSD patients, such as cue-induced fear enhancement [69], abnormal fear memory extinction [160, 161], and negative feedback enhancement of the HPA axis [162-164]. These features of the model show some alterations of PTSD more comprehensively compared to some models caused by a single stimulus [6, 8, 165-168]. However, not all behavioral characteristics of PTSD patients can be accurately recapitulated in animals. For instance, symptoms such as nightmares, self-blame, and trauma-related intrusive thoughts rely heavily on self-reports and subjective experiences, making them difficult to detect in an animal model [10]. These nuanced aspects of PTSD, which are typically assessed through patient self-assessment and feedback, necessitate the development of advanced techniques and tools for detection by researchers. For example, the presence of nightmares could potentially be identified through the use of electrophysiological techniques, similar to those employed in studying sleep fragmentation and disruption [169]. This underscores the need for continuous innovation in research methodologies to bridge the gap between human psychological symptoms and their animal model counterparts.

The susceptibility and resistance to stress in mental illnesses such as depression is an important aspect of studying the pathogenesis [170], and it is also practical for PTSD. Critically, the same traumatic event can yield disparate outcomes in different individuals, prompting much of the research on PTSD to concentrate on susceptibility and individual-specific mechanisms [171, 172]. Nevertheless, systematic interrogation of these mechanisms remains imperative. At present, research in this area mainly focuses on transcriptomic analysis of differential gene expression between locus coeruleus and nucleus cushions [173] and miRNA expression [174]. It is worth noting that changes in microbiota and metabolites are also key mechanisms for stress susceptibility and resistance in SPS models. Researchers conducting genome sequencing on susceptible and resilient mice with PTSD have uncovered significant differences in β-diversity of their gut flora compared to control groups, observed both before and after trauma exposure. Functional analyses revealed that the alterations in the microbiota of susceptible and resilient mice were primarily concentrated on gut-brain interactions and intestinal metabolism. Metabolite assays in the brain further demonstrated abnormally elevated levels of p-cresol, dopamine, and DOPAC in the susceptible mice, whereas resilient mice exhibited a distinctive resilience mechanism associated with myelin formation [171, 175]. The study of susceptibility factors not only provides early insights into the pathophysiology of PTSD but also holds promise for preventive strategies, which are pivotal for effective disease management (Table **3**). Psychological resilience refers to an individual's ability to recover from negative experiences and adapt to their environment after experiencing stress [176]. It is an important factor in the occurrence of stress-related neurological and psychiatric disorders, and also a hot topic in the study of disease mechanisms [177]. Research has shown that the strength of psychological resilience is negatively correlated with the probability of developing PTSD [178]. Clinically, psychological resilience is evaluated using scales, while experimental animal studies classify animals as susceptible and resilient based on behavioral differential analysis to assess psychological resilience [177, 179]. Therefore, conducting research on stress susceptibility and resistance around psychological resilience is also an important approach. Our research group is currently exploring the mechanisms of stress susceptibility and resistance from the perspective of psychological resilience, providing more experimental evidence for the revelation of SPS model mechanisms.

Traumatic stress experiences could engender persistent trauma-associated memories. However, only a minority of exposed individuals develop PTSD symptoms. The SPS model effectively differentiates between resilient and susceptible individuals. SPS induces divergent stress responses in both male and female rodents, with only a subset exhibiting anxiety-like behaviors while others maintain behavioral profiles similar to unstressed controls [184, 185]. Globally, over two-thirds of the population experience traumatic events during their lifetime. While the majority (94.2%) demonstrate resilience without long-term impairment, more than 458 million people develop PTSD symptoms [174, 186, 187]. These findings further validate the reliability of SPS as an animal model of PTSD, as it accurately recapitulates the heterogeneous responses observed in human PTSD populations.

Through modifications of the SPS protocol, specific clinical subpopulations can be precisely modeled. PTSD and attention deficit hyperactivity disorder (ADHD) frequently co-occur, with ADHD known to negatively impact PTSD treatment outcomes [188]. The SPS combined with isolation rearing (IR) paradigm [189] - which maintains IR-induced prepulse inhibition (PPI) deficits under SPS conditions - serves as an excellent model for studying PTSD with comorbid attention deficits (frequently co-occurring with ADHD). Investigations of emotional single prolonged stress (E-SPS) have demonstrated its efficacy in inducing depression/ anxiety-like phenotypes. The E-SPS paradigm also induces alterations in intestinal parameters, producing depression/ anxiety-like behaviors alongside predominant diarrhea-type intestinal dysfunction. This model shows promise for future research on gut-brain interactions and functional gastrointestinal disorders (FGIDs) with psychiatric comorbidities [190]. A study of neonatal isolation (NI) combined with SPS reveals that NI+SPS co-exposure induces more pronounced changes in hippocampal and amygdalar anxiety-related behaviors, spatial memory, and glucocorticoid receptor (GR)/ synaptic protein levels compared to intervention alone [191]. These findings support the “mismatch hypothesis,” which states that early experiences of adversity prepare for the future and promote resilience to similar challenges in later life. That is, if a mismatch occurs between a well-nurtured early life and a traumatic experience in later life, coping ability is affected and vulnerability increases, potentially explaining mechanisms underlying individual differences in PTSD susceptibility [179]. Childhood and adolescent stress exposure represents a significant risk factor for PTSD with comorbid alcohol use disorder (AUD). The adolescent social isolation (SI) plus SPS model has been developed to study PTSD-AUD comorbidity [192]. Collectively, these models provide powerful tools for investigating neurobiological adaptations at the intersection of stress experience, alcohol exposure, biological sex, and mental illness risk factors. These methodological innovations significantly enhance the translational validity of SPS, enabling researchers to dissect the mechanistic foundations specific to distinct PTSD trajectories.

Current preclinical research predominantly focuses on pharmacological agents that serve as prophylactic measures against the onset of disease, a strategy that is inherently logical. Given that PTSD has a relatively high incidence in certain subpopulations (*e.g.* soldiers, police, first responders, *etc.*), preemptive interventions prior to exposure to traumatic events can be effective in forestalling the development of PTSD symptoms. Utilizing the SPS model, researchers have substantiated that numerous pharmacological compounds may possess therapeutic efficacy against PTSD. Some of these compounds are already employed clinically for the treatment of other conditions, while others have yet to be introduced into clinical practice as standalone treatments. Regardless, the majority of these drugs are not currently utilized within the healthcare system for the treatment of PTSD. Therefore, the primary challenge remains to thoroughly investigate these potentially therapeutic agents and transition them into clinical applications.

## CONCLUSION

This review offers significant implications for basic research rooted in the clinical characteristics of PTSD, suggesting that studies utilizing the SPS model could enhance our understanding of PTSD and aid in the identification of effective treatment strategies. The SPS effectively emulates a broad spectrum of PTSD symptoms, making it a valuable tool for researching the pathogenesis and treatment of the disorder. Concurrently, both pharmacological and non-pharmacological treatment strategies derived from this model hold promising translational potential for future clinical applications.

## Figures and Tables

**Table 1:** Comparison of behavior, medical imaging changes and biochemical indices between PTSD patients and SPS model.

**-**	**Patients**	**Animal Model**	**References**
Symptoms and behaviors	Intrusive memories related to the traumatic event. Distress in response to trauma-related cues and avoidance of those cues. Negative alterations in cognition and mood. Increased arousal and reactivity.	Apparent physiological response after exposure to trauma-related stimuli (fear memory: freezing). Avoidance behavior after exposure to trauma reminders (anxiety-like behavior in the elevated cross maze). Sugar preference reduced social interaction, forced-swimming test. Irritability and hypervigilance, startle response, sleep parameter testing.	[1, 15-17]
Brain regions	Decrease in gray matter volume in the hippocampus, amygdala, and prefrontal cortex. Decrease in the cingulum bundle, uncinate fasciculus, increase in the fornix/stria terminalis. Hyperactivation of the amygdala, significant decreases in hippocampal and prefrontal cortex activity, and increases in dorsal anterior cingulate cortex activity.	Increased amygdala activity, decreased function of prefrontal cortex and hippocampus, decreased excitation of prefrontal cortex.	[32, 34-36, 42, 43, 65-68]
Biochemical indicators	Enhanced negative feedback in the HPA axis: reduced cortisol levels, enhanced glucocorticoid receptor (GR) responsiveness and expression. Significant decrease in plasma brain-derived neurotrophic factor (BDNF) concentration; long-term increase in tumor necrosis factor-alpha (TNF-α), interleukin-1 beta (IL-1β) and IL-6 levels. Decreased NAA concentration, increased Glu concentration, and decreased Glu concentration in the anterior cingulate cortex in the hippocampus.	Enhanced negative feedback in the HPA axis and upregulation of GR expression. Increased expression of pro-inflammatory cytokines TNF-α, IL-1β, and IL-6 in the hippocampus. Decrease in Glu concentration and significant increase in striatal N-acetyl-aspartate (NAA) concentration in the medial prefrontal cortex.	[15-17, 51-54, 57-60, 62, 69-72]

**Table 2:** Pre-clinical researches of the therapeutics for PTSD and mechanisms.

**Interventions**		**Mechanisms**	**Results**	**References**
Natural medicines	7,8-Dihydroxyflavone (5 mg/kg/day for 14 days, *i.p.*)	Brain-derived neurotrophic factor (BDNF)-tyrosine kinase receptor B (TrkB) signaling pathway	Prevent astrocytic and synaptic deficits in the hippocampus	[15]
	Korean red ginseng (KRG) (20, 50, or 100 mg/kg/day for 14 days, *i.p.*)	Activate of the serotonergic system	Prevent a reduction in 5-hydroxy tryptamine (5-HT) levels in the brain	[76, 118]
	Anshen Dingzhi prescription (36.8 mg/kg/day for 14 days, *i.g.*)	Mechanistic target of rapamycin (mTOR)-dependent synaptic function	Improve synaptic function in the hippocampus	[23]
	Curculigoside (CUR) (5, 10, 20 mg/kg/day for 14 days, *i.p.*)	Activate cyclic adenosine monophosphate (cAMP)- protein kinase A (PKA) signaling	Rescue hippocampal synaptic deficits	[17]
	Ginsenoside Rg1 (10, 20, 40 mg/kg/day for 14 days, *i.p.*)	Promote synaptic proteins, reducing Kir4.1 and TNF-α in the hippocampus	Promote fear extinction and prevent depression-like behaviors	[16]
	Sibibinin (SIL) (25, 50, and 100 mg/kg/day for 14 days, *i.p.*)	Increased 5-HT levels and enhanced tryptophan hydroxylase (TPH) expression in the hippocampus	Support the anti-depressive and anxiolytic effects	[119]
	Puerarin (50 mg/kg/day for 10 days, *i.g.*)	Normalized biosynthesis of neurosteroids and levels of stress hormones in HPA axis	Support the anti-depressive and anxiolytic effects	[120]
	Tanshinone IIA (TanIIA) (20 mg/kg/day for 7–9 days, *i.p.*)	Activated the CREB/BDNF/TrkB pathway	Ameliorate PTSD-like behaviors	[121]
	Withania somnifera (WS) (500 mg/kg/day, 6 days per week for 6 weeks, *i.g.*)	Preserve changes in antioxidant mechanisms	Antagonize hippocampal oxidative stress	[122]
Chemical medicines	NYX-783 (0.1 mg/kg or 1 mg/kg, 24 h after fear conditioning, *i.p.*)	Positively modulate N-methyl-D-aspartate receptor activity	Reduce fear during extinction	[42]
	(2R,6R)-hydronorketamine (10, 50, and 100 μM, microinjection)	Stimulate GluA1-mediated synaptic plasticity in the PFC	Alleviate anxiety- and depression-like behaviors	[117, 123]
	Neuropeptide Y (2 weeks after SPS, intranasal NPY at 300 µg/rat)	/	Reverse the manifested anxiety and depressive-like behavior	[124]
	The omega-3/6 (1 mg/g, oral gavage daily for 6 days per week for a total of 4 weeks)	Normalized antioxidant mechanisms in the hippocampus	Prevent memory impairment	[92]
	Edaravone (6 mg/kg/day for 21 days, *i.p.*)	Support antioxidant mechanism in the hippocampus	Prevent impairment of short-term and long-term memory	[125]
	Morphine (10 mg/kg, *i.p.*)	The HPA axis and central beta - adrenergic activity	Reverse the SPS-induced deficits in anxiety profile, fear extinction, and dendritic morphology in the medial prefrontal cortex.	[126]
	Aripiprazole (5.0 mg/kg/day for 14 days, *i.p.*)	Agonism of dopamine receptors D2	Reverse the SPS-impaired fear memory dysfunction and the SPS-reduced dopamine efflux in the amygdala.	[127]
	Brain-derived neurotrophic factor (BDNF) (1 mmol, 10 mmol, and 30 mmol/10 μL/day for 7 days, *i.c.v.*)	/	Promote fear extinction and prevented depression-like behaviors	[48]
Non-pharmacological interventions	Acupuncture (Baihui and Yintang, 20 min once per day for 15 days)	Reversed the activation of microglia in the hippocampus, and decreased the expression of IL-1β in serum	Reverse the pathological process of the inflammatory response mediated by the activation of microglia	[128]
	Electroacupuncture (Baihui with a frequency of 2/15 Hz and an intensity of 1 mA for 30 min each day, for 7 days)	Reversed the elevated levels of corticotropin relegating hormone (CRH) and CRHR1 proteins in the amygdala the keap1/nuclear factor erythroid 2-related factor 2 (Nrf2) antioxidant pathway	Upregulate long-term potentiation (LTP) in the hippocampus, repaired synaptic morphology	[129, 130]
	Hyperbaric oxygen therapy (HBOT, 5 days)	Restored the SPS-reduced IL monoamines efflux level and the corticosterone profiles	Restore behaviorally the SPS-impaired fear extinction retrieval ability	[131]
	High intensity interval training (HIIT)	Reduce oxidative stress, anxiety levels, and increasing antioxidant capacity	Protect against neuronal function	[132]

**Table 3:** Biomarkers determine the severity or susceptibility of PTSD.

**Biomarkers**	**Positive (+) or Negative (-) Correlation**	**Aspects of Impact**	**References**
Brain-derived neurotrophic factor (BDNF)	-	Fear extinction	[15, 17, 180]
Glucocorticoid receptor (GR) sensitivity	+	Susceptibility	[56]
Glutamate (Glu)	- (PFC) + (Hip)	Fear memory	[69, 89, 90]
Tumor necrosis factor-alpha (TNF-α), interleukin-1 beta (IL-1β), and interleukin-6 (IL-6)	+	Susceptibility and immune dysregulation	[16, 49, 50]
Corticotropin relegating hormone (CRH), and adrenocorticotropic hormone (ACTH)	+	Anxiolytic	[53, 54]
N-acetyl-aspartate (NAA)	-	Fear memory	[57, 58]
Glutathione (GSH)/oxidized glutathione (GSSG)	-	Memory impairment	[91, 92]
miR-132, miR-153, and miR-142	+	Susceptibility and behavioral impairment	[79-82]
miR-124	-	Behavioral impairment	[83]
Norepinephrine (NE)	+	Contextual processing	[109, 181]
Atrial natriuretic peptide (ANP), and brain natriuretic peptide (BNP)	+	Heart injury	[182]
FK506-binding protein 5 (FKBP5)	+	Susceptibility	[183]
Acetate	-	Susceptibility	[139]

## LIST OF ABBREVIATIONS

7,8-DHF: 7,8-Dihydroxyflavone
ACC: Anterior Cingulate Cortex
ACTH: Adrenocorticotropic Hormone
ADP: Anshen Dingzhi Prescription
AKT: Protein Kinase B
AMY: Amygdala
ANP: Atrial Natriuretic Peptide
AUD: Alcohol Use Disorder
BBB: Blood-brain Barrier
BDNF: Brain-derived Neurotrophic Factor
BNP: Brain Natriuretic Peptide
cAMP: Cyclic Adenosine Monophosphate
CeA: Central Amygdala
CORT: Cortistatin
Cr: Creatine
CRH: Corticotropin Relegating Hormone
eCB: Endocannabinoid
DCC: Deleted in Colorectal Cancer
ERK: Extracellular Signal-regulated Kinase
FS: Foot-shock
GFAP: Glial Fibrillary Acidic Protein
Glu: Glutamate
GPx: Glutathione Peroxidase
GR: Glucocorticoid Receptor
GSH: Glutathione
HCN1: Hyperpolarization-activated Cyclic Nucleotide-gated 1
HIP: Hippocampus
HPA: Hypothalamic-pituitary-adrenal
IL-1β: Interleukin-1 beta
IL-6: Interleukin-6
LC: Locus Coeruleus
mI: Myo-inositol
mTOR: Mechanistic Target of Rapamycin
NAA: N-acetyl-aspartate
NE: Norepinephrine
NET: Norepinephrine Transporter Protein
NF-kB: Nuclear Factor-kappa B
NGF: Nerve Growth Factor
PFC: Prefrontal Cortex
PKA: Protein Kinase A
PPS: Predator-based Psychosocial Stress
PTSD: Post-traumatic Stress Disorder
REM: Rapid Eye Movement
SD: Social Defeat
SPS: Single Prolonged Stress
TBARS: Thiobarbituric Acid Reactive Substances
TNF-α: Tumor Necrosis Factor-alpha
TrkB: Tyrosine Kinase Receptor B

## References

[1] Ressler K.J., Berretta S., Bolshakov V.Y., Rosso I.M., Meloni E.G., Rauch S.L., Carlezon W.A. (2022). Post-traumatic stress disorder: Clinical and translational neuroscience from cells to circuits.. Nat. Rev. Neurol..

[2] Thakur A., Choudhary D., Kumar B., Chaudhary A. (2022). A review on post-traumatic stress disorder (PTSD): Symptoms, therapies and recent case studies.. Curr. Mol. Pharmacol..

[3] Merians A.N., Spiller T., Harpaz-Rotem I., Krystal J.H., Pietrzak R.H. (2023). Post-traumatic stress disorder.. Med. Clin. North Am..

[4] Bisson J.I., Olff M. (2021). Prevention and treatment of PTSD: The current evidence base.. Eur. J. Psychotraumatol..

[5] Török B., Sipos E., Pivac N., Zelena D. (2019). Modelling posttraumatic stress disorders in animals.. Prog. Neuropsychopharmacol. Biol. Psychiatry.

[6] Bouton M.E., Maren S., McNally G.P. (2021). Behavioral and neurobiological mechanisms of pavlovian and instrumental extinction learning.. Physiol. Rev..

[7] Daws S.E., Jamieson S., de Nijs L., Jones M., Snijders C., Klengel T., Joseph N.F., Krauskopf J., Kleinjans J., Vinkers C.H., Boks M.P.M., Geuze E., Vermetten E., Berretta S., Ressler K.J., Rutten B.P.F., Rumbaugh G., Miller C.A. (2020). MicroRNA regulation of persistent stress-enhanced memory.. Mol. Psychiatry.

[8] Van Assche I.A., Padilla M.S., Stupart O.S.R.P., Milton A.L. (2022). Refinement of the stress-enhanced fear learning model of post-traumatic stress disorder: A behavioral and molecular analysis.. Lab Anim. (NY).

[9] Adamec R., Holmes A., Blundell J. (2008). Vulnerability to lasting anxiogenic effects of brief exposure to predator stimuli: Sex, serotonin and other factors—Relevance to PTSD.. Neurosci. Biobehav. Rev..

[10] Schöner J., Heinz A., Endres M., Gertz K., Kronenberg G. (2017). Post‐traumatic stress disorder and beyond: An overview of rodent stress models.. J. Cell. Mol. Med..

[11] Liberzon I., Krstov M., Young E.A. (1997). Stress-restress: Effects on ACTH and fast feedback.. Psychoneuroendocrinology.

[12] Liberzon I., Young E.A. (1997). Effects of stress and glucocorticoids on CNS oxytocin receptor binding.. Psychoneuroendocrinology.

[13] Cathomas F., Murrough J.W., Nestler E.J., Han M.H., Russo S.J. (2019). Neurobiology of resilience: Interface between mind and body.. Biol. Psychiatry.

[14] Almeida F.B., Pinna G., Barros H.M.T. (2021). The role of HPA axis and allopregnanolone on the neurobiology of major depressive disorders and PTSD.. Int. J. Mol. Sci..

[15] Wang J., Gao F., Cui S., Yang S., Gao F., Wang X., Zhu G. (2022). Utility of 7,8-dihydroxyflavone in preventing astrocytic and synaptic deficits in the hippocampus elicited by PTSD.. Pharmacol. Res..

[16] Zhang Z., Song Z., Shen F., Xie P., Wang J., Zhu A., Zhu G. (2021). Ginsenoside Rg1 prevents PTSD-like behaviors in mice through promoting synaptic proteins, reducing Kir4.1 and TNF-α in the Hippocampus.. Mol. Neurobiol..

[17] Ji M., Zhang Z., Gao F., Yang S., Wang J., Wang X., Zhu G. (2023). Curculigoside rescues hippocampal synaptic deficits elicited by PTSD through activating cAMP‐PKA signaling.. Phytother. Res..

[18] Maples-Keller J., Watkins L.E., Nylocks K.M., Yasinski C., Coghlan C., Black K., Jovanovic T., Rauch S.A.M., Rothbaum B.O., Norrholm S.D. (2022). Acquisition, extinction, and return of fear in veterans in intensive outpatient prolonged exposure therapy: A fear-potentiated startle study.. Behav. Res. Ther..

[19] Nahum K., Todder D., Zohar J., Cohen H. (2022). The role of microglia in the (Mal)adaptive response to traumatic experience in an animal model of PTSD.. Int. J. Mol. Sci..

[20] Germain A., Buysse D.J., Nofzinger E. (2008). Sleep-specific mechanisms underlying posttraumatic stress disorder: Integrative review and neurobiological hypotheses.. Sleep Med. Rev..

[21] Clark J.W., Daykin H., Metha J.A., Allocca G., Hoyer D., Drummond S.P.A., Jacobson L.H. (2021). Manipulation of rapid eye movement sleep *via* orexin and GABAA receptor modulators differentially affects fear extinction in mice: Effect of stable versus disrupted circadian rhythm.. Sleep.

[22] Ball T.M., Gunaydin L.A. (2022). Measuring maladaptive avoidance: From animal models to clinical anxiety.. Neuropsychopharmacology.

[23] Yang S., Qu Y., Wang J., Gao F., Ji M., Xie P., Zhu A., Tan B., Wang X., Zhu G. (2022). Anshen Dingzhi prescription in the treatment of PTSD in mice: Investigation of the underlying mechanism from the perspective of hippocampal synaptic function.. Phytomedicine.

[24] Xie P., Chen L., Wang J., Wang X., Yang S., Zhu G. (2024). Polysaccharides from Polygonatum cyrtonema Hua prevent post-traumatic stress disorder behaviors in mice: Mechanisms from the perspective of synaptic injury, oxidative stress, and neuroinflammation.. J. Ethnopharmacol..

[25] Raut S.B., Marathe P.A., van Eijk L., Eri R., Ravindran M., Benedek D.M., Ursano R.J., Canales J.J., Johnson L.R. (2022). Diverse therapeutic developments for post-traumatic stress disorder (PTSD) indicate common mechanisms of memory modulation.. Pharmacol. Ther..

[26] Ding X., Yang M., Wu N., Li J., Song R. (2023). Blockade of dopamine D3 receptor in ventral tegmental area attenuating contextual fear memory.. Biomed. Pharmacother..

[27] Vanderheyden W.M., Lefton M., Flores C.C., Owada Y., Gerstner J.R. (2022). Fabp7 is required for normal sleep suppression and anxiety-associated phenotype following single-prolonged stress in mice.. Neuroglia.

[28] Souza R.R., Noble L.J., McIntyre C.K. (2017). Using the single prolonged stress model to examine the pathophysiology of PTSD.. Front. Pharmacol..

[29] Stander V.A., Thomsen C.J., Highfill-McRoy R.M. (2014). Etiology of depression comorbidity in combat-related PTSD: A review of the literature.. Clin. Psychol. Rev..

[30] Della Valle R., Mohammadmirzaei N., Knox D. (2019). Single prolonged stress alters neural activation in the periacqueductal gray and midline thalamic nuclei during emotional learning and memory.. Learn. Mem..

[31] Deslauriers J., Toth M., Der-Avakian A., Risbrough V.B. (2018). Current status of animal models of posttraumatic stress disorder: Behavioral and biological phenotypes, and future challenges in improving translation.. Biol. Psychiatry.

[32] Harnett N.G., Goodman A.M., Knight D.C. (2020). PTSD-related neuroimaging abnormalities in brain function, structure, and biochemistry.. Exp. Neurol..

[33] Fani N., King T.Z., Shin J., Srivastava A., Brewster R.C., Jovanovic T., Bradley B., Ressler K.J. (2016). Structural and functional connectivity in posttraumatic stress disorder: Associations with FKBP5.. Depress. Anxiety.

[34] Harnett N.G., Ference E.W., Knight A.J., Knight D.C. (2020). White matter microstructure varies with post-traumatic stress severity following medical trauma.. Brain Imaging Behav..

[35] Yehuda R., Hoge C.W., McFarlane A.C., Vermetten E., Lanius R.A., Nievergelt C.M., Hobfoll S.E., Koenen K.C., Neylan T.C., Hyman S.E. (2015). Post-traumatic stress disorder.. Nat. Rev. Dis. Primers.

[36] Acheson D.T., Gresack J.E., Risbrough V.B. (2012). Hippocampal dysfunction effects on context memory: Possible etiology for posttraumatic stress disorder.. Neuropharmacology.

[37] Clancy K.J., Devignes Q., Kumar P., May V., Hammack S.E., Akman E., Casteen E.J., Pernia C.D., Jobson S.A., Lewis M.W., Daskalakis N.P., Carlezon W.A., Ressler K.J., Rauch S.L., Rosso I.M. (2023). Circulating PACAP levels are associated with increased amygdala-default mode network resting-state connectivity in posttraumatic stress disorder.. Neuropsychopharmacology.

[38] Bryant R.A., Felmingham K.L., Malhi G., Andrew E., Korgaonkar M.S. (2021). The distinctive neural circuitry of complex posttraumatic stress disorder during threat processing.. Psychol. Med..

[39] Lanius R.A., Bluhm R., Lanius U., Pain C. (2006). A review of neuroimaging studies in PTSD: Heterogeneity of response to symptom provocation.. J. Psychiatr. Res..

[40] Nicholson A.A., Densmore M., Frewen P.A., Théberge J., Neufeld R.W.J., McKinnon M.C., Lanius R.A. (2015). The dissociative subtype of posttraumatic stress disorder: Unique resting-state functional connectivity of basolateral and centromedial amygdala complexes.. Neuropsychopharmacology.

[41] Zoladz P.R., Diamond D.M. (2013). Current status on behavioral and biological markers of PTSD: A search for clarity in a conflicting literature.. Neurosci. Biobehav. Rev..

[42] Lee B., Pothula S., Wu M., Kang H., Girgenti M.J., Picciotto M.R., DiLeone R.J., Taylor J.R., Duman R.S. (2022). Positive modulation of N-methyl-D-aspartate receptors in the mPFC reduces the spontaneous recovery of fear.. Mol. Psychiatry.

[43] Xiao S., Yang Z., Su T., Gong J., Huang L., Wang Y. (2022). Functional and structural brain abnormalities in posttraumatic stress disorder: A multimodal meta-analysis of neuroimaging studies.. J. Psychiatr. Res..

[44] Domitrovic S.S., Nikolac P.M., Uzun S., Nedic E.G., Kozumplik O., Svob S.D., Mimica N., Pivac N. (2022). Reduced plasma BDNF concentration and cognitive decline in veterans with PTSD.. Psychiatry Res..

[45] Peters J., Dieppa-Perea L.M., Melendez L.M., Quirk G.J. (2010). Induction of fear extinction with hippocampal-infralimbic BDNF.. Science.

[46] Kozlovsky N., Matar M.A., Kaplan Z., Kotler M., Zohar J., Cohen H. (2007). Long-term down-regulation of BDNF mRNA in rat hippocampal CA1 subregion correlates with PTSD-like behavioural stress response.. Int. J. Neuropsychopharmacol..

[47] Takei S., Morinobu S., Yamamoto S., Fuchikami M., Matsumoto T., Yamawaki S. (2011). Enhanced hippocampal BDNF/TrkB signaling in response to fear conditioning in an animal model of posttraumatic stress disorder.. J. Psychiatr. Res..

[48] Yin J.B., Liu H.X., Shi W., Ding T., Hu H.Q., Guo H.W., Jin S., Wang X.L., Zhang T., Lu Y.C., Cao B.Z. (2022). Various BDNF administrations attenuate SPS-induced anxiety-like behaviors.. Neurosci. Lett..

[49] Passos I.C., Vasconcelos-Moreno M.P., Costa L.G., Kunz M., Brietzke E., Quevedo J., Salum G., Magalhães P.V., Kapczinski F., Kauer-Sant’Anna M. (2015). Inflammatory markers in post-traumatic stress disorder: A systematic review, meta-analysis, and meta-regression.. Lancet Psychiatry.

[50] Imai R., Hori H., Itoh M., Lin M., Niwa M., Ino K., Ogawa S., Ishida M., Sekiguchi A., Matsui M., Kunugi H., Akechi T., Kamo T., Kim Y. (2018). Inflammatory markers and their possible effects on cognitive function in women with posttraumatic stress disorder.. J. Psychiatr. Res..

[51] Daskalakis N.P., Cohen H., Nievergelt C.M., Baker D.G., Buxbaum J.D., Russo S.J., Yehuda R. (2016). New translational perspectives
for blood-based biomarkers of PTSD: From glucocorticoid to
immune mediators of stress susceptibility. Exp. Neurol.,.

[52] Hendrickson R.C., Raskind M.A. (2016). Noradrenergic dysregulation in the pathophysiology of PTSD. Exp. Neurol.,.

[53] Yahyavi S.T., Zarghami M., Naghshvar F., Danesh A. (2015). Relationship of cortisol, norepinephrine, and epinephrine levels with war-induced posttraumatic stress disorder in fathers and their offspring.. Rev. Bras. Psiquiatr..

[54] Delahanty D.L., Raimonde A.J., Spoonster E. (2000). Initial posttraumatic urinary cortisol levels predict subsequent PTSD symptoms in motor vehicle accident victims.. Biol. Psychiatry.

[55] Agorastos A., Kellner M., Baker D.G., Otte C. (2014). When time stands still.. Curr. Opin. Psychiatry.

[56] Mellman T.A., Bustamante V., Fins A.I., Pigeon W.R., Nolan B. (2002). REM sleep and the early development of posttraumatic stress disorder.. Am. J. Psychiatry.

[57] Ham B.J., Chey J., Yoon S.J., Sung Y., Jeong D.U., Ju Kim S., Sim M.E., Choi N., Choi I.G., Renshaw P.F., Lyoo I.K. (2007). Decreased N‐acetyl‐aspartate levels in anterior cingulate and hippocampus in subjects with post‐traumatic stress disorder: A proton magnetic resonance spectroscopy study.. Eur. J. Neurosci..

[58] Knox D., Perrine S.A., George S.A., Galloway M.P., Liberzon I. (2010). Single prolonged stress decreases glutamate, glutamine, and creatine concentrations in the rat medial prefrontal cortex.. Neurosci. Lett..

[59] Chen S., Lin Z., Tan K.L., Chen R., Su W., Zhao H., Tan Q., Tan W. (2020). Enhanced contextual fear memory and elevated astroglial glutamate synthase activity in hippocampal CA1 BChE shRNA knockdown mice.. Front. Psychiatry.

[60] Fumagalli F., Pasini M., Frasca A., Drago F., Racagni G., Riva M.A. (2009). Prenatal stress alters glutamatergic system responsiveness in adult rat prefrontal cortex.. J. Neurochem..

[61] Ousdal O.T., Milde A.M., Craven A.R., Ersland L., Endestad T., Melinder A., Huys Q.J., Hugdahl K. (2019). Prefrontal glutamate levels predict altered amygdala–prefrontal connectivity in traumatized youths.. Psychol. Med..

[62] Su X., Xia C., Wang W., Sun H., Tan Q., Zhang S., Li L., Kemp G.J., Yue Q., Gong Q. (2018). Abnormal metabolite concentrations and amygdala volume in patients with recent-onset posttraumatic stress disorder.. J. Affect. Disord..

[63] McDonald A.J., Mott D.D. (2017). Functional neuroanatomy of amygdalohippocampal interconnections and their role in learning and memory.. J. Neurosci. Res..

[64] Fang Q., Li Z., Huang G.D., Zhang H.H., Chen Y.Y., Zhang L.B., Ding Z.B., Shi J., Lu L., Yang J.L. (2018). Traumatic stress produces distinct activations of GABAergic and glutamatergic neurons in Amygdala.. Front. Neurosci..

[65] Misaki M., Mulyana B., Zotev V., Wurfel B.E., Krueger F., Feldner M., Bodurka J. (2021). Hippocampal volume recovery with real-time functional MRI amygdala neurofeedback emotional training for posttraumatic stress disorder.. J. Affect. Disord..

[66] Fragkaki I., Thomaes K., Sijbrandij M. (2016). Posttraumatic stress disorder under ongoing threat: A review of neurobiological and neuroendocrine findings.. Eur. J. Psychotraumatol..

[67] Nelson M.D., Tumpap A.M. (2017). Posttraumatic stress disorder symptom severity is associated with left hippocampal volume reduction: A meta-analytic study.. CNS Spectr..

[68] Smith B.M., Thomasson M., Yang Y.C., Sibert C., Stocco A. (2021). When fear shrinks the brain: A computational model of the effects of posttraumatic stress on hippocampal volume.. Top. Cogn. Sci..

[69] Perrine S.A., Eagle A.L., George S.A., Mulo K., Kohler R.J., Gerard J., Harutyunyan A., Hool S.M., Susick L.L., Schneider B.L., Ghoddoussi F., Galloway M.P., Liberzon I., Conti A.C. (2016). Severe, multimodal stress exposure induces PTSD-like characteristics in a mouse model of single prolonged stress.. Behav. Brain Res..

[70] Matsumoto Y., Morinobu S., Yamamoto S., Matsumoto T., Takei S., Fujita Y., Yamawaki S. (2013). Vorinostat ameliorates impaired fear extinction possibly *via* the hippocampal NMDA-CaMKII pathway in an animal model of posttraumatic stress disorder.. Psychopharmacology.

[71] Liu F., Yang L., Sun X., Zhang H., Pan W., Wang X., Yang J., Ji M., Yuan H. (2016). NOX2 mediated-parvalbumin interneuron loss might contribute to anxiety-like and enhanced fear learning behavior in a rat model of post-traumatic stress disorder.. Mol. Neurobiol..

[72] Murray S.L., Holton K.F. (2021). Post-traumatic stress disorder may set the neurobiological stage for eating disorders: A focus on glutamatergic dysfunction.. Appetite.

[73] Pittenger C., Duman R.S. (2008). Stress, depression, and neuroplasticity: A convergence of mechanisms.. Neuropsychopharmacology.

[74] Yang S., Hu J., Chen Y., Zhang Z., Wang J., Zhu G. (2024). DCC, a potential target for controlling fear memory extinction and hippocampal LTP in male mice receiving single prolonged stress.. Neurobiol. Stress.

[75] Sun J., Jia K., Sun M., Zhang X., Chen J., Zhu G., Li C., Lian B., Du Z., Sun H., Sun L. (2022). The GluA1-related BDNF pathway is involved in PTSD-induced cognitive flexibility deficit in attentional set-shifting tasks of rats.. J. Clin. Med..

[76] Lee B., Sur B., Oh S. (2022). Neuroprotective effect of Korean Red Ginseng against single prolonged stress-induced memory impairments and inflammation in the rat brain associated with BDNF expression.. J. Ginseng Res..

[77] Ehrlich I., Klein M., Rumpel S., Malinow R. (2007). PSD-95 is required for activity-driven synapse stabilization.. Proc. Natl. Acad. Sci. USA.

[78] Zhang L., Deng L., Ma C., Zhang H., Dang Y. (2023). Brain-Derived neurotrophic factor delivered intranasally relieves post-traumatic stress disorder symptoms caused by a single prolonged stress in rats.. Neuropsychobiology.

[79] Chen Y.L., Tong L., Chen Y., Fu C.H., Peng J.B., Ji L.L. (2022). miR-153 downregulation alleviates PTSD-like behaviors and reduces cell apoptosis by upregulating the Sigma-1 receptor in the hippocampus of rats exposed to single-prolonged stress.. Exp. Neurol..

[80] Tong L., Li M.D., Nie P.Y., Chen Y., Chen Y.L., Ji L.L. (2021). miR-132 downregulation alleviates behavioral impairment of rats exposed to single prolonged stress, reduces the level of apoptosis in PFC, and upregulates the expression of MeCP2 and BDNF.. Neurobiol. Stress.

[81] Nie P.Y., Ji L.L., Fu C.H., Peng J.B., Wang Z.Y., Tong L. (2021). miR-132 regulates PTSD-like behaviors in rats following single-prolonged stress through Fragile X-related protein 1.. Cell. Mol. Neurobiol..

[82] Ji L.L., Ye Y., Nie P.Y., Peng J.B., Fu C.H., Wang Z.Y., Tong L. (2019). Dysregulation of miR-142 results in anxiety-like behaviors following single prolonged stress.. Behav. Brain Res..

[83] Chen Y., An Q., Yang S.T., Chen Y.L., Tong L., Ji L.L. (2022). MicroRNA-124 attenuates PTSD-like behaviors and reduces the level of inflammatory cytokines by downregulating the expression of TRAF6 in the hippocampus of rats following single-prolonged stress.. Exp. Neurol..

[84] Chang X., Wang J., Jiang H., Shi L., Xie J. (2019). Hyperpolarization-activated cyclic nucleotide-gated channels: An emerging role in neurodegenerative diseases.. Front. Mol. Neurosci..

[85] Ni L., Xu Y., Dong S., Kong Y., Wang H., Lu G., Wang Y., Li Q., Li C., Du Z., Sun H., Sun L. (2020). The potential role of the HCN1 ion channel and BDNF-mTOR signaling pathways and synaptic transmission in the alleviation of PTSD.. Transl. Psychiatry.

[86] Zhang X., Zhao Y., Du Y., Sun H., Zhang W., Wang A., Li Q., Li C., Wang Y., Du Z., Sun H., Sun L. (2021). Effect of ketamine on mood dysfunction and spatial cognition deficits in PTSD mouse models *via* HCN1–BDNF signaling.. J. Affect. Disord..

[87] Wen Y., Li B., Han F., Wang E., Shi Y. (2012). Dysfunction of calcium/calmodulin/CaM kinase IIα cascades in the medial prefrontal cortex in post-traumatic stress disorder.. Mol. Med. Rep..

[88] Zheng S., Han F., Shi Y., Wen L., Han D. (2017). Single-prolonged-stress-induced changes in autophagy-related proteins Beclin-1, LC3, and p62 in the medial prefrontal cortex of rats with post-traumatic stress disorder.. J. Mol. Neurosci..

[89] George S.A., Rodriguez-Santiago M., Riley J., Rodriguez E., Liberzon I. (2015). The effect of chronic phenytoin administration on single prolonged stress induced extinction retention deficits and glucocorticoid upregulation in the rat medial prefrontal cortex.. Psychopharmacology.

[90] Rosso I.M., Crowley D.J., Silveri M.M., Rauch S.L., Jensen J.E. (2017). Hippocampus glutamate and N-Acetyl aspartate markers of excitotoxic neuronal compromise in posttraumatic stress disorder.. Neuropsychopharmacology.

[91] Coimbra-Costa D., Alva N., Duran M., Carbonell T., Rama R. (2017). Oxidative stress and apoptosis after acute respiratory hypoxia and reoxygenation in rat brain.. Redox Biol..

[92] Alquraan L., Alzoubi K.H., Hammad H., Rababa’h S.Y., Mayyas F. (2019). Omega-3 fatty acids prevent post-traumatic stress disorder-induced memory impairment.. Biomolecules.

[93] Araki M., Fuchikami M., Omura J., Miyagi T., Nagashima N., Okamoto Y., Morinobu S. (2020). The role of glucocorticoid receptors in the induction and prevention of hippocampal abnormalities in an animal model of posttraumatic stress disorder.. Psychopharmacology.

[94] Li B., Zhang D., Verkhratsky A. (2022). Astrocytes in post-traumatic stress disorder.. Neurosci. Bull..

[95] Ney L.J., Crombie K.M., Mayo L.M., Felmingham K.L., Bowser T., Matthews A. (2022). Translation of animal endocannabinoid models of PTSD mechanisms to humans: Where to next?. Neurosci. Biobehav. Rev..

[96] Gunduz-Cinar O., Castillo L.I., Xia M., Van Leer E., Brockway E.T., Pollack G.A., Yasmin F., Bukalo O., Limoges A., Oreizi-Esfahani S., Kondev V., Báldi R., Dong A., Harvey-White J., Cinar R., Kunos G., Li Y., Zweifel L.S., Patel S., Holmes A. (2023). A cortico-amygdala neural substrate for endocannabinoid modulation of fear extinction.. Neuron.

[97] Xie G., Gao X., Guo Q., Liang H., Yao L., Li W., Ma B., Wu N., Han X., Li J. (2024). Cannabidiol ameliorates PTSD-like symptoms by inhibiting neuroinflammation through its action on CB2 receptors in the brain of male mice.. Brain Behav. Immun..

[98] Xue F., Xue S., Liu L., Sang H., Ma Q., Tan Q., Wang H., Zhou C., Peng Z. (2019). Early intervention with electroacupuncture prevents PTSD-like behaviors in rats through enhancing hippocampal endocannabinoid signaling.. Prog. Neuropsychopharmacol. Biol. Psychiatry.

[99] Jeon M., Kim M.S., Kong C.H., Min H.S., Kang W.C., Park K., Jung S.Y., Bae H.J., Park S.J., Lee J.Y., Kim J.W., Ryu J.H. (2025). 4-Methoxycinnamic acid ameliorates post-traumatic stress disorder-like behavior in mice by antagonizing the CRF type 1 receptor.. Life Sci..

[100] Zhu J., Wang C., wang Y., Guo C., Lu P., Mou F., Shao S. (2022). Electroacupuncture alleviates anxiety and modulates amygdala CRH/CRHR1 signaling in single prolonged stress mice.. Acupunct. Med..

[101] Tillinger A., Zvozilová A., Mach M., Horváthová Ľ., Dziewiczová L., Osacká J. (2024). Single intranasal administration of Ucn3 affects the development of PTSD symptoms in an animal model.. Int. J. Mol. Sci..

[102] Wang Z., Hu X., Wang Z., Chen J., Wang L., Li C., Deng J., Yue K., Wang L., Kong Y., Sun L. (2024). Ketamine alleviates PTSD-like effect and improves hippocampal synaptic plasticity *via* regulation of GSK-3β/GR signaling of rats.. J. Psychiatr. Res..

[103] Wang S.C., Lin C.C., Tzeng N.S., Tung C.S., Liu Y.P. (2019). Effects of oxytocin on prosocial behavior and the associated profiles of oxytocinergic and corticotropin-releasing hormone receptors in a rodent model of posttraumatic stress disorder.. J. Biomed. Sci..

[104] Frijling J.L. (2017). Preventing PTSD with oxytocin: Effects of oxytocin administration on fear neurocircuitry and PTSD symptom development in recently trauma-exposed individuals.. Eur. J. Psychotraumatol..

[105] Wang S.C., Lin C.C., Chen C.C., Tzeng N.S., Liu Y.P. (2018). Effects of oxytocin on fear memory and neuroinflammation in a rodent model of posttraumatic stress disorder.. Int. J. Mol. Sci..

[106] Le Dorze C., Borreca A., Pignataro A., Ammassari-Teule M., Gisquet-Verrier P. (2020). Emotional remodeling with oxytocin durably rescues trauma-induced behavioral and neuro-morphological changes in rats: A promising treatment for PTSD.. Transl. Psychiatry.

[107] Eskandarian S., Vafaei A.A., Vaezi G.H., Taherian F., Kashefi A., Rashidy-Pour A. (2013). Effects of systemic administration of oxytocin on contextual fear extinction in a rat model of post-traumatic stress disorder.. Basic Clin. Neurosci..

[108] Li M., Han F., Shi Y. (2011). Expression of locus coeruleus mineralocorticoid receptor and glucocorticoid receptor in rats under single-prolonged stress.. Neurol. Sci..

[109] Hou Y., Li M., Jin Y., Xu F., Liang S., Xue C., Wang K., Zhao W. (2021). Protective effects of tetramethylpyrazine on dysfunction of the locus coeruleus in rats exposed to single prolonged stress by anti-ER stress mechanism.. Psychopharmacology.

[110] George S.A., Knox D., Curtis A.L., Aldridge J.W., Valentino R.J., Liberzon I. (2013). Altered locus coeruleus–norepinephrine function following single prolonged stress.. Eur. J. Neurosci..

[111] Nwokafor C., Serova L.I., Tanelian A., Nahvi R.J., Sabban E.L. (2021). Variable response of norepinephrine transporter to traumatic stress and relationship to hyperarousal.. Front. Behav. Neurosci..

[112] Salehabadi S., Abrari K., Elahdadi Salmani M., Nasiri M., Lashkarbolouki T. (2020). Investigating the role of the amygdala orexin receptor 1 in memory acquisition and extinction in a rat model of PTSD.. Behav. Brain Res..

[113] Han D., Han F., Shi Y., Zheng S., Wen L. (2020). Mechanisms of memory impairment induced by Orexin-A *via* Orexin 1 and Orexin 2 receptors in post-traumatic stress disorder rats.. Neuroscience.

[114] Kong C.H., Min H.S., Jeon M., Kang W.C., Park K., Kim M.S., Jung S.Y., Bae H.J., Park S.J., Shin H.K., Seo C.S., Ryu J.H. (2024). Cheonwangbosimdan mitigates post-traumatic stress disorder-like behaviors through GluN2B-containing NMDA receptor antagonism in mice.. J. Ethnopharmacol..

[115] Traylor M., Persyn E., Tomppo L., Klasson S., Abedi V., Bakker M.K., Torres N., Li L., Bell S., Rutten-Jacobs L., Tozer D.J., Griessenauer C.J., Zhang Y., Pedersen A., Sharma P., Jimenez-Conde J., Rundek T., Grewal R.P., Lindgren A., Meschia J.F., Salomaa V., Havulinna A., Kourkoulis C., Crawford K., Marini S., Mitchell B.D., Kittner S.J., Rosand J., Dichgans M., Jern C., Strbian D., Fernandez-Cadenas I., Zand R., Ruigrok Y., Rost N., Lemmens R., Rothwell P.M., Anderson C.D., Wardlaw J., Lewis C.M., Markus H.S., Helsinki Stroke S.D.P.I-C.A.S.G. (2021). Genetic basis of lacunar stroke: A pooled analysis of individual patient data and genome-wide association studies.. Lancet Neurol..

[116] Riggs L.M., Aracava Y., Zanos P., Fischell J., Albuquerque E.X., Pereira E.F.R., Thompson S.M., Gould T.D. (2020). (2R,6R)-hydroxynorketamine rapidly potentiates hippocampal glutamatergic transmission through a synapse-specific presynaptic mechanism.. Neuropsychopharmacology.

[117] Li Y., Du Y., Wang C., Lu G., Sun H., Kong Y., Wang W., Lian B., Li C., Wang L., Zhang X., Sun L. (2022). (2R,6R)-hydroxynorketamine acts through GluA1-induced synaptic plasticity to alleviate PTSD-like effects in rat models.. Neurobiol. Stress.

[118] Lee B., Sur B., Lee H., Oh S. (2020). Korean Red Ginseng prevents posttraumatic stress disorder–triggered depression-like behaviors in rats *via* activation of the serotonergic system.. J. Ginseng Res..

[119] Lee B., Choi G.M., Sur B. (2020). Silibinin prevents depression-like behaviors in a single prolonged stress rat model: The possible role of serotonin.. BMC Complement. Med. Ther..

[120] Su A., Chen X., Zhang Z., Xu B., Wang C., Xu Z. (2021). Integrated transcriptomic and metabolomic analysis of rat serum to investigate potential target of puerarin in the treatment post-traumatic stress disorder.. Ann. Transl. Med..

[121] Jiang Y., Wang X., Li X., Liu A., Fan Q., Yang L., Feng B., Zhang K., Lu L., Qi J., Yang F., Song D., Wu Y., Zhao M., Liu S. (2022). Tanshinone IIA improves contextual fear‐ and anxiety‐like behaviors in mice *via* the CREB/BDNF/TrkB signaling pathway.. Phytother. Res..

[122] Alzoubi K.H., Al Hilo A.S., Al-Balas Q.A., El-Salem K., El-Elimat T., Alali F.Q. (2019). Withania somnifera root powder protects againist post-traumatic stress disorder-induced memory impairment.. Mol. Biol. Rep..

[123] Gou L., Li Y., Liu S., Sang H., Lan J., Chen J., Wang L., Li C., Lian B., Zhang X., Sun H., Sun L. (2023). (2R,6R)-hydroxynorketamine improves PTSD-associated behaviors and structural plasticity *via* modulating BDNF-mTOR signaling in the nucleus accumbens.. J. Affect. Disord..

[124] Serova L.I., Nwokafor C., Van Bockstaele E.J., Reyes B.A.S., Lin X., Sabban E.L. (2019). Single prolonged stress PTSD model triggers progressive severity of anxiety, altered gene expression in locus coeruleus and hypothalamus and effected sensitivity to NPY.. Eur. Neuropsychopharmacol..

[125] Alzoubi K.H., Shatnawi A., Al-Qudah M.A., Alfaqih M.A., (2 and 3-Spec Issue), 201-207. (2019). Edaravone prevents memory impairment in an animal model of post-traumatic distress.Behav. Pharmacol.,.

[126] Abdullahi P.R., Raeis-Abdollahi E., Sameni H., Vafaei A.A., Ghanbari A., Rashidy-Pour A. (2020). Protective effects of morphine in a rat model of post-traumatic stress disorder: Role of hypothalamic-pituitary-adrenal axis and beta- adrenergic system.. Behav. Brain Res..

[127] Lin C.C., Chang H.A., Tai Y.M., Chen T.Y., Wan F.J., Chang C.C., Tung C.S., Liu Y.P. (2019). Subchronic administration of aripiprazole improves fear extinction retrieval of Pavlovian conditioning paradigm in rats experiencing psychological trauma.. Behav. Brain Res..

[128] Jiang H., Chen L., Li Y., Gao X., Yang X., Zhao B., Li Y., Wang Y., Yu X., Zhang X., Feng S., Chai Y., Meng H., Ren X., Bao T. (2023). Effects of acupuncture on regulating the hippocampal inflammatory response in rats exposed to post-traumatic stress disorder.. Neurosci. Lett..

[129] Zhou C.H., Xue F., Shi Q.Q., Xue S.S., Zhang T., Ma X.X., Yu L.S., Liu C., Wang H.N., Peng Z.W. (2022). The impact of electroacupuncture early intervention on the brain lipidome in a mouse model of post-traumatic stress disorder.. Front. Mol. Neurosci..

[130] Hou Y., Chen M., Wang C., Liu L., Mao H., Qu X., Shen X., Yu B., Liu S. (2021). Electroacupuncture attenuates anxiety-like behaviors in a rat model of post-traumatic stress disorder: The role of the ventromedial prefrontal cortex.. Front. Neurosci..

[131] Lin C.C., Huang K.L., Tung C.S., Liu Y.P. (2019). Hyperbaric oxygen therapy restored traumatic stress-induced dysregulation of fear memory and related neurochemical abnormalities.. Behav. Brain Res..

[132] Koyuncuoğlu T., Sevim H., Çetrez N., Meral Z., Gönenç B., Kuntsal Dertsiz E., Akakın D., Yüksel M., Çakır Ö.K. (2021). High intensity interval training protects from Post Traumatic Stress Disorder induced cognitive impairment.. Behav. Brain Res..

[133] Badour C.L., Flores J., Hood C.O., Jones A.C., Brake C.A., Tipsword J.M., Penn C.J., McCann J.P. (2023). Concurrent and proximal associations among PTSD symptoms, prescription opioid use, and co-use of other substances: Results from a daily monitoring study.. Psychol. Trauma.

[134] Rodríguez M.N., Colgan D.D., Leyde S., Pike K., Merrill J.O., Price C.J. (2024). Trauma exposure across the lifespan among individuals engaged in treatment with medication for opioid use disorder: Differences by gender, PTSD status, and chronic pain.. Subst. Abuse Treat. Prev. Policy.

[135] Peck K.R., Badger G.J., Cole R., Higgins S.T., Moxley-Kelly N., Sigmon S.C. (2023). Prolonged exposure therapy for PTSD in individuals with opioid use disorder: A randomized pilot study.. Addict. Behav..

[136] Petrakis I.L., Meshberg-Cohen S., Nich C., Kelly M.M., Claudio T., Jane J.S., Pisani E., Ralevski E. (2024). Cognitive processing therapy (CPT) versus individual drug counseling (IDC) for PTSD for veterans with opioid use disorder maintained on buprenorphine.. Am. J. Addict..

[137] Fuchs-Leitner I., Yazdi K., Gerstgrasser N.W., Tholen M.G., Graffius S.T., Schorb A., Rosenleitner J. (2021). Risk of PTSD due to the COVID-19 pandemic among patients in opioid substitution treatment.. Front. Psychiatry.

[138] Tanelian A., Nankova B., Miari M., Nahvi R.J., Sabban E.L. (2022). Resilience or susceptibility to traumatic stress: Potential influence of the microbiome.. Neurobiol. Stress.

[139] Tanelian A., Nankova B., Hu F., Sahawneh J.D., Sabban E.L. (2023). Effect of acetate supplementation on traumatic stress-induced behavioral impairments in male rats.. Neurobiol. Stress.

[140] Chen L., Zhang Y., Wang Z., Zhang Z., Wang J., Zhu G., Yang S. (2024). Activation of GPER1 by G1 prevents PTSD ‐like behaviors in mice: Illustrating the mechanisms from BDNF/TrkB to mitochondria and synaptic connection.. CNS Neurosci. Ther..

[141] Gao F., Wang J., Yang S., Ji M., Zhu G. (2023). Fear extinction induced by activation of PKA ameliorates anxiety-like behavior in PTSD mice.. Neuropharmacology.

[142] Chen D., Wang J., Cao J., Zhu G. (2024). cAMP-PKA signaling pathway and anxiety: Where do we go next?. Cell. Signal..

[143] Su A., Zhang J., Zou J. (2019). The anxiolytic-like effects of puerarin on an animal model of PTSD.. Biomed. Pharmacother..

[144] Cui J.J., Huang Z.Y., Xie Y.H., Wu J.B., Xu G.H., Li C.F., Zhang M.M., Yi L.T. (2023). Gut microbiota mediated inflammation, neuroendocrine and neurotrophic functions involved in the antidepressant-like effects of diosgenin in chronic restraint stress.. J. Affect. Disord..

[145] Malik H., Usman M., Arif M., Ahmed Z., Ali G., Rauf K., Sewell R.D.E. (2023). Diosgenin normalization of disrupted behavioral and central neurochemical activity after single prolonged stress.. Front. Pharmacol..

[146] Chen X.D., Wei J.X., Wang H.Y., Peng Y.Y., Tang C., Ding Y., Li S., Long Z.Y., Lu X.M., Wang Y.T. (2023). Effects and mechanisms of salidroside on the behavior of SPS-induced PTSD rats.. Neuropharmacology.

[147] Hu J., Li H., Wang X., Cheng H., Zhu G., Yang S. (2024). Novel mechanisms of Anshen Dingzhi prescription against PTSD: Inhibiting DCC to modulate synaptic function and inflammatory responses.. J. Ethnopharmacol..

[148] Wang J., Zhao P., Cheng P., Zhang Z., Yang S., Wang J., Wang X., Zhu G. (2024). Exploring the effect of Anshen Dingzhi prescription on hippocampal mitochondrial signals in single prolonged stress mouse model.. J. Ethnopharmacol..

[149] Shen F., Song Z., Xie P., Li L., Wang B., Peng D., Zhu G. (2021). Polygonatum sibiricum polysaccharide prevents depression-like behaviors by reducing oxidative stress, inflammation, and cellular and synaptic damage.. J. Ethnopharmacol..

[150] Shen F., Xie P., Li C., Bian Z., Wang X., Peng D., Zhu G. (2022). Polysaccharides from Polygonatum cyrtonema Hua reduce depression-like behavior in mice by inhibiting oxidative stress-Calpain-1-NLRP3 signaling axis.. Oxid. Med. Cell. Longev..

[151] Li M., Li K., Zhang H., Jiang Y. (2019). Study on the mechanism of TMRK electroacupuncture in repairing synaptic plasticity in amygdala and hippocampus to relieve fear memory in PTSD rats.. Technol. Health Care.

[152] Zhou C., Xue F., Xue S., Sang H., Liu L., Wang Y., Cai M., Zhang Z.J., Tan Q., Wang H., Peng Z. (2019). Electroacupuncture pretreatment ameliorates PTSD-like behaviors in rats by enhancing hippocampal neurogenesis *via* the Keap1/Nrf2 antioxidant signaling pathway.. Front. Cell. Neurosci..

[153] Chen X., Liu C., Du K., Chen Y., Yang S., Zhu G., Wang J. (2024). Effects and mechanisms of electroacupuncture on fear extinction and sleep phase in single prolonged stress mice.. Zhongguo Zhenjiu.

[154] Salehpour F., Mahmoudi J., Kamari F., Sadigh-Eteghad S., Rasta S.H., Hamblin M.R. (2018). Brain photobiomodulation therapy: A narrative review.. Mol. Neurobiol..

[155] Zhao C., Li D., Kong Y., Liu H., Hu Y., Niu H., Jensen O., Li X., Liu H., Song Y. (2022). Transcranial photobiomodulation enhances visual working memory capacity in humans.. Sci. Adv..

[156] Li Y., Dong Y., Yang L., Tucker L., Yang B., Zong X., Hamblin M.R., Zhang Q. (2021). Transcranial photobiomodulation prevents PTSD-like comorbidities in rats experiencing underwater trauma.. Transl. Psychiatry.

[157] Li Y., Dong Y., Yang L., Tucker L., Zong X., Brann D., Hamblin M.R., Vazdarjanova A., Zhang Q. (2021). Photobiomodulation prevents PTSD-like memory impairments in rats.. Mol. Psychiatry.

[158] Zhao H., Li Y., Luo T., Chou W., Sun T., Liu H., Qiu H., Zhu D., Chen D., Gu Y. (2023). Preventing Post-Traumatic Stress Disorder (PTSD) in rats with pulsed 810 nm laser transcranial phototherapy.. Transl. Psychiatry.

[159] Jung J.T.K., Marques L.S., Zborowski V.A., Silva G.L., Nogueira C.W., Zeni G. (2023). Resistance training modulates hippocampal neuroinflammation and protects anxiety-depression-like dyad induced by an emotional single prolonged stress model.. Mol. Neurobiol..

[160] Chang S.H., Chen H.Y., Shaw F.Z., Shyu B.C. (2023). Early- and late-phase changes of brain activity and early-phase neuromodulation in the posttraumatic stress disorder rat model.. Neurobiol. Stress.

[161] Han F., Jiang J., Ding J., Liu H., Xiao B., Shi Y. (2017). Change of Rin1 and Stathmin in the animal model of traumatic stresses.. Front. Behav. Neurosci..

[162] Szeszko P.R., Lehrner A., Yehuda R. (2018). Glucocorticoids and hippocampal structure and function in PTSD.. Harv. Rev. Psychiatry.

[163] Fischer S., Schumacher T., Knaevelsrud C., Ehlert U., Schumacher S. (2021). Genes and hormones of the hypothalamic-pituitary-adrenal axis in post-traumatic stress disorder. What is their role in symptom expression and treatment response?. J. Neural Transm..

[164] Ganon-Elazar E., Akirav I. (2012). Cannabinoids prevent the development of behavioral and endocrine alterations in a rat model of intense stress.. Neuropsychopharmacology.

[165] Zambetti P.R., Schuessler B.P., Lecamp B.E., Shin A., Kim E.J., Kim J.J. (2022). Ecological analysis of Pavlovian fear conditioning in rats.. Commun. Biol..

[166] Jones M.E., Lebonville C.L., Paniccia J.E., Balentine M.E., Reissner K.J., Lysle D.T. (2018). Hippocampal interleukin-1 mediates stress-enhanced fear learning: A potential role for astrocyte-derived interleukin-1β.. Brain Behav. Immun..

[167] Matsukawa M., Yoshikawa M., Katsuyama N., Aizawa S., Sato T. (2022). The anterior piriform cortex and predator odor responses: Modulation by inhibitory circuits.. Front. Behav. Neurosci..

[168] Tyler R.E., Weinberg B.Z.S., Lovelock D.F., Ornelas L.C., Besheer J. (2020). Exposure to the predator odor TMT induces early and late differential gene expression related to stress and excitatory synaptic function throughout the brain in male rats.. Genes Brain Behav..

[169] Philbert J., Beeské S., Belzung C., Griebel G. (2015). The CRF1 receptor antagonist SSR125543 prevents stress-induced long-lasting sleep disturbances in a mouse model of PTSD: Comparison with paroxetine and d-cycloserine.. Behav. Brain Res..

[170] Cheng C.M., Chen M.H., Tsai S.J., Chang W.H., Tsai C.F., Lin W.C., Bai Y.M., Su T.P., Chen T.J., Li C.T. (2024). Susceptibility to treatment-resistant depression within families.. JAMA Psychiatry.

[171] Tseilikman V.E., Tseilikman O.B., Pashkov A.A., Ivleva I.S., Karpenko M.N., Shatilov V.A., Zhukov M.S., Fedotova J.O., Kondashevskaya M.V., Downey H.F., Manukhina E.B. (2022). Mechanisms of susceptibility and resilience to PTSD: Role of dopamine metabolism and BDNF expression in the hippocampus.. Int. J. Mol. Sci..

[172] Alexander K.S., Nalloor R., Bunting K.M., Vazdarjanova A. (2020). Investigating individual pre-trauma susceptibility to a PTSD-like phenotype in animals.. Front. Syst. Neurosci..

[173] Nahvi R.J., Tanelian A., Nwokafor C., Godino A., Parise E., Estill M., Shen L., Nestler E.J., Sabban E.L. (2023). Transcriptome profiles associated with resilience and susceptibility to single prolonged stress in the locus coeruleus and nucleus accumbens in male sprague-dawley rats.. Behav. Brain Res..

[174] Huang G., Iqbal J., Shen D., Xue Y., Yang M., Jia X. (2023). MicroRNA expression profiles of stress susceptibility and resilience in the prelimbic and infralimbic cortex of rats after single prolonged stress.. Front. Psychiatry.

[175] Laudani S., Torrisi S.A., Alboni S., Bastiaanssen T.F.S., Benatti C., Rivi V., Moloney R.D., Fuochi V., Furneri P.M., Drago F., Salomone S., Tascedda F., Cryan J.F., Leggio G.M. (2023). Gut microbiota alterations promote traumatic stress susceptibility associated with p-cresol-induced dopaminergic dysfunctions.. Brain Behav. Immun..

[176] Troy A.S., Willroth E.C., Shallcross A.J., Giuliani N.R., Gross J.J., Mauss I.B. (2023). Psychological resilience: An affect-regulation framework.. Annu. Rev. Psychol..

[177] Li H.Y., Zhu M.Z., Yuan X.R., Guo Z.X., Pan Y.D., Li Y.Q., Zhu X.H. (2023). A thalamic-primary auditory cortex circuit mediates resilience to stress.. Cell.

[178] Rakesh G., Morey R.A., Zannas A.S., Malik Z., Marx C.E., Clausen A.N., Kritzer M.D., Szabo S.T. (2019). Resilience as a translational endpoint in the treatment of PTSD.. Mol. Psychiatry.

[179] Willmore L., Cameron C., Yang J., Witten I.B., Falkner A.L. (2022). Behavioural and dopaminergic signatures of resilience.. Nature.

[180] Zhang X.X., Tian Y., Wang Z.T., Ma Y.H., Tan L., Yu J.T. (2021). The epidemiology of Alzheimer’s disease modifiable risk factors and prevention.. J. Prev. Alzheimers Dis..

[181] Seo D., Zhang E.T., Piantadosi S.C., Marcus D.J., Motard L.E., Kan B.K., Gomez A.M., Nguyen T.K., Xia L., Bruchas M.R. (2021). A locus coeruleus to dentate gyrus noradrenergic circuit modulates aversive contextual processing.. Neuron.

[182] Liu M., Xie J., Sun Y. (2020). TLR4/MyD88/NF-κB-mediated inflammation contributes to cardiac dysfunction in rats of PTSD.. Cell. Mol. Neurobiol..

[183] Sun J., Yu X., Huangpu H., Yao F. (2019). Ginsenoside Rb3 protects cardiomyocytes against hypoxia/reoxygenation injury *via* activating the antioxidation signaling pathway of PERK/Nrf2/HMOX1.. Biomed. Pharmacother..

[184] Le Dorze C., Gisquet-Verrier P. (2016). Sensitivity to trauma-associated cues is restricted to vulnerable traumatized rats and reinstated after extinction by yohimbine.. Behav. Brain Res..

[185] Tanelian A., Nankova B., Cheriyan A., Arens C., Hu F., Sabban E.L. (2023). Differences in gut microbiota associated with stress resilience and susceptibility to single prolonged stress in female rodents.. Neurobiol. Stress.

[186] Javidi H., Yadollahie M. (2012). Post-traumatic stress disorder.. Int. J. Occup. Environ. Med..

[187] Mayou R., Bryant B., Ehlers A. (2001). Prediction of psychological outcomes one year after a motor vehicle accident.. Am. J. Psychiatry.

[188] Wang Z., Zuschlag Z.D., Myers U.S., Hamner M. (2022). Atomoxetine in comorbid ADHD/PTSD: A randomized, placebo controlled, pilot, and feasibility study.. Depress. Anxiety.

[189] Lin C.C., Chen T.Y., Cheng P.Y., Liu Y.P. (2020). Early life social experience affects adulthood fear extinction deficit and associated dopamine profile abnormalities in a rat model of PTSD.. Prog. Neuropsychopharmacol. Biol. Psychiatry.

[190] Marques L.S., Jung J.T.K., Zborowski V.A., Pinheiro R.C., Nogueira C.W., Zeni G. (2023). Emotional-Single Prolonged Stress: A promising model to illustrate the gut-brain interaction.. Physiol. Behav..

[191] Cheng W., Han F., Shi Y. (2019). Neonatal isolation modulates glucocorticoid-receptor function and synaptic plasticity of hippocampal and amygdala neurons in a rat model of single prolonged stress.. J. Affect. Disord..

[192] Pitcairn S.R., Ortelli O.A., Weiner J.L. (2024). Effects of early social isolation and adolescent single prolonged stress on anxiety‐like behaviors and voluntary ethanol consumption in female long evans rats.. Alcohol. Clin. Exp. Res..

